# Women’s income and risk of intimate partner violence: secondary findings from the MAISHA cluster randomised trial in North-Western Tanzania

**DOI:** 10.1186/s12889-019-7454-1

**Published:** 2019-08-14

**Authors:** Tanya Abramsky, Shelley Lees, Heidi Stöckl, Sheila Harvey, Imma Kapinga, Meghna Ranganathan, Gerry Mshana, Saidi Kapiga

**Affiliations:** 1 Department of Global Health and Development, London School of Hygiene and Tropical Medicine, 15-17 Tavistock Place, London, WC1H 9SH UK; 2 Mwanza Intervention Trials Unit, PO Box 11936, Mwanza, Tanzania; 3 Department of Infectious Diseases Epidemiology, London School of Hygiene and Tropical Medicine, Keppel Street, London, WC1E 7HT UK; 4 National Institute for Medical Research, Isamilo Road, Mwanza, Tanzania

**Keywords:** Intimate partner violence, Economic abuse, Economic empowerment, Income, Tanzania, Women, Africa

## Abstract

**Background:**

Intimate partner violence (IPV) is pervasive throughout the world, with profound consequences for women’s health. While women’s ‘economic empowerment’ is touted as a potential means to reduce IPV, evidence is mixed as to the role of different economic factors in determining women’s risk. This paper explores associations and potential pathways between women’s income and experience of IPV, in Mwanza city, Tanzania.

**Methods:**

We use data from married/cohabiting women (*N* = 740) enrolled in the MAISHA study, a cluster randomised trial of an IPV prevention intervention. Women were interviewed at baseline and 29-months later. We use logistic regression to model cross-sectional (baseline) and longitudinal associations between: a woman’s monthly income (quartiles) and her past year risk of physical IPV, sexual IPV and economic abuse; and a woman’s relative financial contribution to the household (same/less than partner; more than partner) and past year physical IPV and sexual IPV.

**Results:**

At baseline, 96% of respondents reported earning an income and 28% contributed more financially to the household than their partner did. Higher income was associated with lower past-year physical IPV risk at baseline and longitudinally, and lower sexual IPV at baseline only. No clear associations were seen between income and economic abuse. Higher relative financial contribution was associated with increased physical IPV and sexual IPV among all women at baseline, though only among control women longitudinally. Higher income was associated with several potential pathways to reduced IPV, including reduced household hardship, fewer arguments over the partner’s inability to provide for the family, improved relationship dynamics, and increased relationship dissolution. Those contributing more than their partner tended to come from more disadvantaged households, argue more over their partner’s inability to provide, and have worse relationship dynamics.

**Conclusions:**

While women’s income was protective against IPV, women who contributed more financially than their partners had greater IPV risk. Poverty and tensions over men’s inability to provide emerge as potentially important drivers of this association. Interventions to empower women should not only broaden women’s access to economic resources and opportunities, but also work with women and men to address men’s livelihoods, male gender roles and masculinity norms.

**Trial registration:**

ClinicalTrials.gov #NCT02592252, registered retrospectively (13/08/2015).

**Electronic supplementary material:**

The online version of this article (10.1186/s12889-019-7454-1) contains supplementary material, which is available to authorized users.

## Background

Violence against women is a public health and human rights problem that is pervasive throughout the world. Recent estimates suggest that one third of women globally have experienced physical and/or sexual violence by a partner during their lifetime [1]. The consequences of violence are far reaching for women’s mental and physical health [2–4], their participation in social and economic activity, and the health, education and well-being of their children [5].

In recent years, interventions that seek to economically ‘empower’ women have been identified as a potential means through which to reduce women’s risk of intimate partner violence (IPV). Microfinance-based interventions, cash transfer programmes, and other forms of livelihoods programming have in some cases and in some settings been shown to reduce women’s risk of IPV [6–9]. Posited mechanisms through which such interventions may work [8] include: (i) increased economic security leading to reduced poverty-related stress, improved mental health, and concomitant reductions in men’s alcohol use and aggression [7, 10]; (ii) increased household cash flow reducing conflict over money and resources; and (iii) a shift in relationship power dynamics resulting from the woman’s increased financial and social confidence, her increased capacity to leave (or threaten to leave) a relationship, and her partner’s increased appreciation of her worth [11, 12].

However, the evidence on whether such interventions work to empower women and reduce IPV risk is mixed [6, 13]. Several studies from Bangladesh have produced results variously showing microcredit membership to be associated with decreased IPV risk [14, 15], increased IPV risk [16–19] particularly in certain conservative or urban contexts, or no change in IPV risk [20]. Other studies suggest an initial increase in risk followed by a decrease in the longer term [21–23]. Microfinance combined with a gender training intervention was associated with a 55% decrease in relative risk of past year physical and/or sexual IPV among women in rural South Africa [24], though when offered without the gender training component was not associated with the same benefits [25]. Other interventions combining microfinance or village savings and loans schemes with a gender transformative component (such as training or couples discussion groups) have also been associated with reductions in IPV [26–28]. A recent review of cash transfer programmes [8] showed cash transfers to be associated with decreases in IPV in 11 out of the 14 included studies, though the authors highlighted that impacts may depend on programme design features (for example whether combined with other intervention components) and contextual factors influencing how the interventions are received by participants and their partners.

The picture from observational studies examining the links between a woman’s economic circumstances and her current IPV risk is similarly complex [7]. For example, women’s employment or working for money has been associated with lower violence in some settings [29] but higher in others [19, 29–32] with some suggestion that formal employment may be more protective than irregular or seasonal employment [33] and longer duration of employment more protective than shorter [34]. Other studies from a range of countries indicate no association between women’s employment or income and IPV [19, 29, 35]. Women’s higher economic contribution to the household was associated with higher past year physical violence in one study in Bangladesh [15], but no significant association was found in two other Bangladesh sites [14, 21] or in the Philippines [36].

It is now widely accepted that associations between a woman’s economic circumstances and IPV risk differ according to the economic indicator used [37], type of violence [38], and setting, though empirical evidence to explain this variation and unpack mechanisms of association is limited [7]. The increase in risk that has been observed in some settings has been attributed to a ‘male-backlash’ – as women gain more economic autonomy, men who feel that their authority is being challenged may increase their use of violence as a means of reasserting their control [31, 39, 40]. Some posit that whether or not such a backlash occurs may depend in large part on social norms, with evidence from Bangladesh suggesting that microcredit programmes are associated with increased violence in more conservative settings, but not in more progressive settings [18]. It is hypothesised that in settings where women don’t commonly work outside the home, their entry into work may initially increase marital tensions and risk of IPV, but over time lead to decreased IPV risk (as social norms about the acceptability of women’s employment change and men recognise the benefits of additional household income) [7]. This theory is supported by cross-country analysis comparing IPV risk among women working in settings where many women work and those where few women work [41].

It is likely too, that multiple pathways operate, in sometimes opposite directions, at the same time – for example where increased financial independence allows a woman to negotiate change within (or leave) an abusive relationship, but is also perceived by her partner as a threat to his status as provider [42].

In addition to more ‘absolute’ measures of a woman’s economic situation, are measures comparing a woman’s occupational or income status to that of her partner. A multi-country study conducted by the WHO found that in 6 out of 14 sites women who work but have partners who don’t are at increased risk of past year physical/sexual IPV, compared to a situation where both he and she work. However, in some other sites associations in the opposite direction were observed [43]. Similarly, status inconsistencies in educational attainment (where the woman has higher education than her partner) have been associated with increased risk of IPV in some settings but decreases in others [7, 43]. It is likely that in some contexts such status inconsistencies represent a threat to men’s status as head of the household (in line with relative resource theory) [44, 45], while in contexts where the partner has more egalitarian views they are not perceived as such [46].

Since interventions such as microfinance or cash transfers for women have the potential to introduce such disparities in financial contribution, it is important to understand when a woman’s higher financial contribution to the household is associated with increased risk of IPV and when it is not. This would aid in the development of complementary intervention components to counteract potential harms arising from economic interventions.

In this paper, we use data from married/cohabiting women enrolled in the MAISHA study [47], in Mwanza city, Tanzania, to further explore the relationship between a woman’s income and her risk of physical IPV, sexual IPV and economic abuse. We use two measures of the woman’s economic status: (a) her reported income, categorised into quartiles; and (b) her financial contribution to the household relative to that of her partner’s.

In order to better understand the nature and mechanisms of these associations, we:

- Describe how individual-, partner-, and household-level characteristics vary between married or cohabiting women (a) in different income quartiles, and (b) whose financial contribution to the household is more than that of their partner, versus the same/less.
- Explore cross-sectional and longitudinal associations between (a) the woman’s income and her past year risk of physical IPV, sexual IPV and economic abuse by a partner; and (b) the woman’s relative financial contribution to the household and her past year risk of physical IPV and sexual IPV.
- Explore potential pathways through which these economic variables may influence a woman’s risk of physical IPV, sexual IPV and economic abuse.
- Examine similarities/differences in observed associations between women receiving a social empowerment intervention in addition to the microfinance, and women just receiving microfinance.

## Methods

### Study design

We conducted a cluster randomised trial (CRT) to assess the impact of an IPV prevention intervention on empowerment, and health and IPV-related outcomes, among women already enrolled in a microfinance programme. Full details of the study and MAISHA intervention have been reported elsewhere [47]. Briefly, we recruited 66 established BRAC (Bangladesh Rural Advancement Committee) microfinance loan groups from low socio-economic communities in Mwanza city. Each loan group comprises about 10–30 women, and loans are administered at this level. Individual-level written informed consent was obtained from all women before they could participate in the study, though refusal to participate had no bearing on their continued involvement with BRAC. Following completion of baseline data collection, loan groups were randomly allocated in a 1:1 ratio to either continue with microfinance only (*N* = 33) or to receive a social empowerment intervention in addition to microfinance (*N* = 33). The additional intervention included 10 sessions delivered by female facilitators over 20 weeks.

Women were interviewed, using a structured questionnaire, at baseline and 2 years after completion of the intervention (29 months post baseline). This secondary analysis uses data from both the baseline and follow-up surveys. The interviews were conducted face-to-face in Swahili, and responses were entered directly onto a tablet computer programmed to check for accuracy and consistency of entered data. Interviews were conducted in a private location, by female interviewers trained on interviewing techniques, gender issues, violence, and ethical issues related to IPV research [48, 49]. The questionnaires (Additional files 1 and 2) were developed specifically for this study and included questions on household details, income, relationships, health, childhood and experiences of specific acts of IPV and abuse.

Income was measured by asking women how much they earn (in Tanzanian Shillings) on a typical working day/week/month. Reported daily and weekly earnings were converted to monthly earnings on the assumption that each participant worked for 22 days per month. Monthly earnings were then coded into quartiles, with higher quartile indicating higher income. Separate codes were created for women not earning and those who reported not knowing how much they earned. A woman’s financial contribution to the household relative to her husband’s/partner’s, was measured with the question: “Would you say that the money you bring into the household is more than what your husband/partner contributes, less than what he contributes, or about the same as he contributes?” The outcome was recoded as binary: same/less than partner; more than partner.

Questions on IPV were adapted from the WHO (World Health Organization) Violence Against Women instrument [50]. Briefly, women’s past year experiences of physical and sexual IPV were measured using questions on experiences of specific violent acts by a partner. If they answered yes to having experienced any physically violent act (ranging from being slapped to having weapons used against them) they were classified as having experienced physical IPV, and if they reported experiencing any sexually violent act (including having sexual intercourse when they did not want to because they were afraid of what their partner might do if they refused) they were classified as having experienced sexual IPV. A similar approach was used to measure economic abuse, with items measuring male partners’ behaviours that restrict the woman’s access to economic resources (money for household expenses and money she has earned) and exclude her from participation in financial decision making (see Additional file 3 for more details). Respondents who reported having experienced violence or abuse were provided with information/referrals to support services within their communities.

The study was conducted in accordance with WHO recommendations on researching violence against women [48], and received ethical approval from the Tanzanian National Health Research Ethics Committee of the National Institute for Medical Research (Ref: NIMR/HQ/R.8a/Vol.IX/1512), and the ethics committee of the London School of Hygiene and Tropical Medicine (Ref: 11642).

### Conceptual framework for this paper

Both income quartile and relative financial contribution were considered as exposures in relation to physical and sexual IPV. Only income quartile was considered as an exposure for economic abuse, as there was too much overlap in our measure of economic abuse and relative financial contribution to allow meaningful exploration of an association between the two (economic abuse included the male partner refusing to give the woman enough money for household expenses even when he had money for other things). All women in the study were participating in a microfinance programme, and thus the impact of microfinance on risk of abuse could not be explored in this paper.

**Fig. 1:**
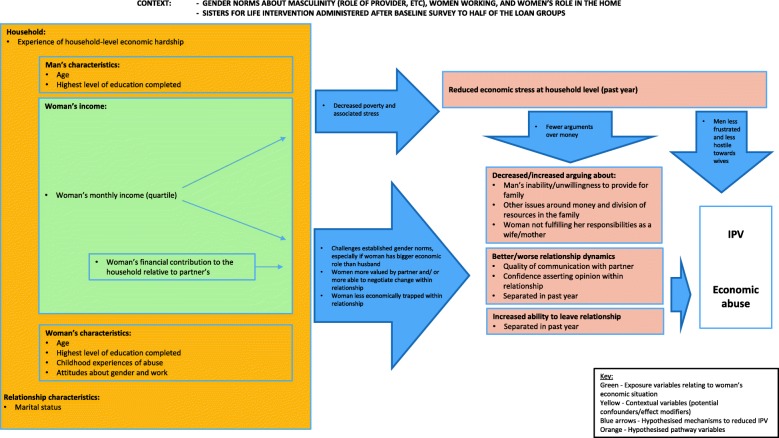
Conceptual framework of the association between a woman’s income and past year experience of IPV

The context and potential pathways between a woman’s income and her risk of IPV/economic abuse are outlined in Fig. 1. This framework acknowledges the possibility that a woman’s income can influence her risk of IPV in either direction and through multiple pathways: (a) decreasing economic stress in the household (and consequent economic vulnerability experienced by her partner); (b) constituting transgression of a gender/social norm; (c) making her more valued by her partner and/or more confident to negotiate change in her relationship; and (d) providing her with the financial confidence to leave (or threaten to leave) an abusive relationship. Measured indicators of more proximate triggers of IPV/economic abuse that lie on these pathways include: decreased economic stress at the household-level; decreased or increased arguing about gender roles and money issues; improved or worsened relationship dynamics; and increased levels of separation. A woman’s absolute income is hypothesised to influence IPV risk through all four pathways, and her financial contribution relative to her husband’s through pathways (b), (c) and (d). Different pathways may operate in opposite directions simultaneously, thereby reducing any net effect of income on IPV risk.

The relationship between a woman’s income and risk of physical and sexual IPV/economic abuse may also be explained (confounded) or modified by contextual factors. These include factors operating at the household-level (household economic hardship, if women contribute more *because* their partners are low earners), relationship-level (duration of relationship), individual woman-level (age, education, gender attitudes, childhood experiences of abuse), and individual-man level (age, education). We also consider how women’s participation in a social empowerment intervention (as measured at the loan group level) may modify the association between her income and IPV risk.

The framework does not lay out an exhaustive list of contextual or pathway factors that may be important in understanding the association between income and IPV, as we are restricted by data availability. We lack, for example, data on partner’s attitudes related to gender roles and specific community-wide norms such as the acceptability of women working for money that could be important modifiers of the association.

### Statistical analysis

The analysis was restricted to married or cohabiting women (for whom relative contribution to the household is a relevant measure). All analyses using just baseline (cross-sectional) data were pooled across intervention and control arm women as they were not hypothesised to differ in any way prior to intervention implementation. All analyses using baseline and follow-up (longitudinal) data were performed separately for intervention and control arm women, as we hypothesised that involvement in the social empowerment intervention could modify the association between income and abuse.

First, we generated baseline descriptive data on women, their partners and households, disaggregated by (i) the woman’s income quartile and (ii) her relative financial contribution to the household.

Age-adjusted odds ratios (OR) of association between income indicators and physical IPV, sexual IPV and economic abuse were estimated using logistic regression. Two age-adjusted logistic regression models were fitted for each exposure/outcome relationship (i) to estimate the cross-sectional association between baseline measure of exposure and baseline measure of outcome, and (ii) to estimate the association between change in exposure (between baseline and follow-up) and follow-up measure of the outcome. We then produced a multivariate model for each outcome that included the economic exposure indicator(s) (both income and relative financial contribution for physical IPV and sexual IPV; and income only for economic abuse), and potential confounders (individual-, partner, and household-level contextual factors). For each outcome, we tested for interactions between the economic indicator(s) and the woman’s age, education, household hardship, and her beliefs over whether the man should be the primary provider for the family.

Baseline cross-sectional associations between the income indicators and potential pathway variables were explored through cross-tabulations. We used logistic regression to calculate age-adjusted odds ratios between the potential pathway variables (see Additional file 3) and IPV.

## Results

One thousand and forty-nine women were enrolled into the trial, of whom 1021 (97%) completed a baseline survey (September 2014 – June 2015). Seven hundred and forty women were married/living as married at the time of the survey and were thus included in this cross-sectional analysis of baseline data. 919 (90%) women went on to complete the follow-up survey (May – December 2017), 587 of whom were married/living as married at both baseline and follow-up and so included in the longitudinal analysis.

Overall, 709 (96%) women in our sample who were currently married or living as married were earning an income at baseline, and 208 (28%) reported contributing more financially to the household than their husband did (versus less or the same) (Table 1). Among those earning an income, there was no significant association between the woman’s monthly income quartile and her financial contribution to the household relative to her husband’s contribution.

**Table 1:** Characteristics of married/cohabiting women, disaggregated by income quartile and relative financial contribution to household

	Total	Relative financial contribution to household		Woman’s monthly income					
		Smaller/same financial contribution to household as husband	Greater financial contribution to household than husband	Doesn’t earn	1st quartile (lowest)	2nd quartile	3rd quartile	4th quartile (highest)	Don’t know
Total		533/740 (72%)	207/740 (28%)	31 (4%)	206 (28%)	199 (27%)	145 (20%)	132 (18%)	27 (4%)
Exposure variable									
Woman’s monthly income									
Doesn’t earn	31	31 (100%)	0 (0%)	–	–	–	–	–	–
1st quartile (lowest)	206	145 (70%)	61 (30%)	–	–	–	–	–	–
2nd quartile	199	151 (76%)	48 (24%)	–	–	–	–	–	–
3rd quartile	145	107 (74%)	38 (26%)	–	–	–	–	–	–
4th quartile (highest)	132	83 (63%)	49 (37%)	–	–	–	–	–	–
Don’t know	27	16 (59%)	11 (41%)	–	–	–	–	–	–
Woman’s characteristics									
Age									
< 30 years	118	101 (86%)	17 (15%)	7 (6%)	37 (31%)	32 (27%)	22 (19%)	19 (16%)	1 (1%)
30–39 years	325	246 (76%)	79 (24%)	10 (3%)	87 (27%)	83 (26%)	65 (20%)	65 (20%)	15 (5%)
40–49 years	209	135 (65%)	74 (35%)	9 (4%)	60 (29%)	60 (29%)	38 (18%)	34 (16%)	8 (4%)
50+ years	88	51 (58%)	37 (42%)	5 (6%)	22 (25%)	24 (27%)	20 (23%)	14 (16%)	3 (3%)
Education									
None/incomplete primary	102	66 (65%)	36 (35%)	9 (9%)	28 (27%)	25 (25%)	16 (16%)	19 (19%)	5 (5%)
Completed primary	491	362 (74%)	129 (26%)	19 (4%)	152 (31%)	126 (26%)	101 (21%)	79 (16%)	14 (3%)
Attended secondary or higher	147	105 (71%)	42 (29%)	3 (2%)	26 (18%)	48 (33%)	28 (19%)	34 (23%)	8 (5%)
Believes man should be primary provider									
No	220	155 (70%)	65 (30%)	10 (5%)	48 (22%)	63 (29%)	38 (17%)	51 (23%)	10 (5%)
Yes	520	378 (73%)	142 (27%)	21 (4%)	158 (30%)	136 (26%)	107 (21%)	81 (16%)	17 (3%)
Witnessed violence against a parent/household member in the home as a child									
Never	266	198 (74%)	68 (26%)	12 (5%)	75 (28%)	76 (29%)	44 (17%)	48 (18%)	11 (4%)
Once/few times	260	193 (74%)	67 (26%)	11 (4%)	66 (25%)	69 (27%)	59 (23%)	43 (17%)	12 (5%)
Many times	214	142 (66%)	72 (34%)	8 (4%)	65 (30%)	54 (25%)	42 (20%)	41 (19%)	4 (2%)
Partner’s characteristics									
Partner’s Age									
< 40	205	171 (83%)	34 (17%)	13 (6%)	59 (29%)	46 (22%)	45 (22%)	39 (19%)	3 (1%)
40–49	264	196 (74%)	68 (26%)	6 (2%)	74 (28%)	74 (28%)	53 (20%)	47 (18%)	10 (4%)
50+	239	153 (64%)	86 (36%)	12 (5%)	61 (26%)	75 (31%)	41 (17%)	41 (17%)	9 (4%)
Don’t know	32	13 (41%)	19 (59%)	0 (0%)	12 (38%)	4 (13%)	6 (19%)	5 (16%)	5 (16%)
Partner’s education									
Completed primary or below/don’t know	468	327 (70%)	141 (30%)	24 (5%)	148 (32%)	127 (27%)	80 (17%)	72 (15%)	17 (4%)
Above primary	272	206 (76%)	66 (24%)	7 (3%)	58 (21%)	72 (26%)	65 (24%)	60 (22%)	10(4%)
How often she has seen him drunk in past year									
Never/partner doesn’t drink	504	376 (75%)	128 (25%)	21 (4%)	133 (26%)	139 (28%)	98 (19%)	97 (19%)	16 (3%)
Once/few times	101	74 (73%)	27 (27%)	7 (7%)	31 (31%)	26 (26%)	24 (24%)	10 (10%)	3 (3%)
Many	133	83 (62%)	50 (38%)	3 (2%)	42 (32%)	33 (25%)	22 (17%)	25 (19%)	8 6%)
Relationship level									
Relationship duration									
< 5 years	62	46 (74%)	16 (26%)	3 (5%)	16 (26%)	14 (23%)	11 (18%)	14 (23%)	4 (6%)
5–9.99 years	101	71 (70%)	30 (30%)	4 (4%)	29 (29%)	26 (26%)	24 (24%)	17 (17%)	1 (1%)
10+ years	577	416 (72%)	161 (28%)	24 (4%)	161 (28%)	159 (28%)	110 (19%)	101 (18%)	22 (4%)
Household-level									
Experienced economic hardship in past year									
No	432	339 (78%)	93 (22%)	19 (4%)	107 (25%)	107 (25%)	96 (22%)	88 (20%)	15 (3%)
Yes	308	1947 (63%)	114 (37%)	12 (4%)	99 (32%)	92 (30%)	49 (16%)	44 (14%)	12 (4%)

### How do individual-, partner- and household-level characteristics differ according to women’s income and relative financial contribution to the household? (Table 1)

Women’s income was not systematically related to age, though older women were more likely than younger women to contribute more to the household than their partner (42% of those in the oldest age-group versus 15% of those in the youngest age-group). Similar patterns were seen in relation to partner’s age where it was known.

Secondary/higher education (woman’s and partner’s) was associated with the woman’s higher income, but decreased likelihood that she contributed more than her partner.

Women’s income tended to be lower among those who believed that a man should be the primary provider for the family, but her relative financial contribution to the household was not associated with this belief.

A woman’s income was not associated with whether or not she had witnessed violence against a household member in her home as a child, though those who had witnessed violence many times were slightly more likely to report contributing more financially than their partner.

Women who had seen their husband drunk many times in the past year were more likely than those whose partners were drunk a few times/never to contribute more financially than their partners. However, frequency of partner’s drunkenness was not associated with the woman’s monthly income per se.

Women living in households that had experienced economic hardship (insufficient resources to meet basic necessities) in the past year were more likely than those in households without economic hardship to be in the lower income quartiles (1st or 2nd) but to contribute more than their husband to household finances (37% versus 22%).

### Is a woman’s income/relative financial contribution related to her past year risk of physical IPV, sexual IPV, economic abuse?

#### Past year physical IPV (Tables 2 and 3)

At baseline, those in the higher income quartiles were less likely to experience physical IPV compared to women in the lowest income quartile, an association that remained though weakened in the multivariate model. However, risk of physical IPV was lowest amongst those not working at all (adjusted odds ratio (aOR) comparing those not working to those in lowest quartile: 0.24, 95% confidence interval (CI) 0.08–0.69). In intervention groups, women who had moved up at least one income quartile or started working between baseline and follow-up were less likely to report IPV at follow-up compared to women who had experienced a decrease in income quartile or stopped working during that time (aOR 0.39, 95%CI 0.17–0.89). In control groups, a similar pattern was observed though the association was weaker.

**Table 2:** Cross-sectional association between woman’s income and past year experience of physical IPV, sexual IPV and economic abuse at baseline^a^ (*n* = 740)

	Past year physical IPV at baseline			Past year sexual IPV at baseline			Past year economic abuse at baseline		
	n/N (%)	Age-adjusted OR (95%CI)	aOR^c^ (95%CI)	n/N (%)	Age-adjusted OR (95%CI)	aOR^c^ (95%CI)	n/N (%)	Age-adjusted OR (95%CI)	aOR^d^ (95%CI)
Monthly income^b^									
1st quartile (< 60 USD)	64/206 (31%)	–	–	49/206 (24%)	–	–	77/206 (37%)	–	–
2nd quartile (60–119 USD)	46/199 (23%)	0.68 (0.46–1.00)	0.74 (0.49–1.12)	40/199 (20%)	0.82 (0.50–1.36)	0.88 (0.53–1.46)	88/199 (44%)	1.33 (0.91–1.96)	1.46 (0.97–2.19)
3rd quartile (120–234 USD)	23/145 (16%)	0.42 (0.27–0.65)	0.47 (0.30–0.75)	19/145 (13%)	0.50 (0.28–0.89)	0.54 (0.29–0.98)	38/145 (26%)	0.60 (0.36–1.01)	0.67 (0.39–1.16)
4th quartile (> 235 USD)	24/132 (18%)	0.48 (0.28–0.84)	0.56 (0.32–0.98)	22/132 (17%)	0.65 (0.34–1.25)	0.71 (0.37–1.39)	43/132 (33%)	0.81 (0.50–1.32)	0.95 (0.57–1.59)
Doesn’t know	7/27 (26%)	0.85 (0.36–2.03)	0.95 (0.35–2.55)	7/27 (26%)	1.24 (0.52–2.95)	1.57 (0.60–4.15)	14/27 (52%)	1.79 (0.87–3.71)	2.25 (1.02–4.98)
Doesn’t earn	3/31 (10%)	0.23 (0.08–0.66)	0.24 (0.08–0.69)	5/31 (16%)	0.61 (0.22–1.67)	0.63 (0.22–1.81)	11/31 (35%)	0.95 (0.43–2.09)	1.12 (0.43–2.88)
Financial contribution to household relative to husband’s									
He higher/same	111/533 (21%)	–	–	94/533 (18%)	–	–			
She higher	56/207 (27%)	1.83 (1.23–2.71)	1.52 (0.97–2.38)	48/207 (23%)	1.65 (1.09–2.52)	1.43 (0.88–2.34)			

^a^Among women currently married/living as married at baseline

^b^Participants reported their earnings in Tanzanian shillings in terms of either daily, weekly or monthly income. Reported daily and weekly earnings were converted to monthly earnings on the assumption that each participant worked for 22 days per month. The conversion to US Dollars (USD) was based on the exchange rate at the time of data collection (1 USD = 1887 Tanzanian shillings)

^c^Adjusted for woman’s age, other income/financial contribution variable, partner’s age, having witnessed IPV as a child, woman’s education, partner’s education, relationship duration, and experience of household-level financial hardship in past year

^d^Adjusted for woman’s age, partner’s age, having witnessed IPV as a child, woman’s education, partner’s education, relationship duration, and experience of household-level financial hardship in past year

**Table 3:** Longitudinal association between woman’s income and past year experience of physical IPV^a^

	Intervention arm (*n* = 313)			Control arm (*n* = 274)		
	Past year physical IPV at follow-up n/N (%)	Age-adjusted OR (95%CI)	aOR^b^ (95%CI)	Past year physical IPV at follow-up n/N (%)	Age-adjusted OR (95%CI)	aOR^b^ (95%CI)
Change in monthly income between baseline and follow-up						
Fallen 1+ quartile/stopped working	22/86 (26%)	–	–	17/77 (22%)	–	–
Stayed in same quartile	16/96 (17%)	0.63 (0.30–1.32)	0.48 (0.19–1.22)	19/78 (24%)	1.18 (0.57–2.43)	1.04 (0.51–2.14)
Increased 1+ quartile/started working	16/112 (14%)	0.52 (0.29–0.94)	0.39 (0.17–0.89)	18/99 (18%)	0.76 (0.38–1.51)	0.55 (0.25–1.23)
Change in financial contribution to household relative to husband between baseline and follow-up						
Was always lower/the same	37/195 (19%)	–	–	28/161 (17%)	–	–
Was higher, now lower/same	5/33 (15%)	0.85 (0.28–2.54)	0.83 (0.23–2.98)	4/30 (13%)	0.93 (0.30–2.87)	0.81 (0.22–2.91)
Was lower/same, now higher	5/45 (11%)	0.58 (0.25–1.30)	0.87 (0.37–2.05)	12/39 (31%)	2.80 (1.06–7.38)	4.16 (1.44–12.01)
Always higher	8/40 (20%)	1.37 (0.64–2.93)	1.84 (0.65–5.25)	17/44 (39%)	4.46 (1.85–10.77)	4.76 (1.84–12.34)

^a^Among women married/living as married at baseline and follow-up

^b^Adjusted for woman’s age, other income/financial contribution variable, partner’s age, baseline measure of outcome, woman’s education, partner’s education, relationship duration, and experience of household-level financial hardship in past year

At baseline, those whose financial contribution to the household exceeded that of their partner had a higher risk of physical IPV (aOR 1.52, 95%CI 0.97–2.38), compared to those contributing the same/less. A similar pattern was seen in the control arm at follow-up, where highest risk of physical IPV was observed among those who had consistently contributed more financially than their husband (at both baseline and follow-up); next highest risk among those who had newly begun contributing more (at follow-up but not at baseline); and lowest risk among women who consistently or newly contributed less than/the same as their husband. This association was not observed in the intervention arm at follow-up.

There was no strong evidence of interactions between either of the economic variables and the woman’s age or past year household economic hardship. There was some suggestion that the association between higher financial contribution and physical IPV was stronger among those attending secondary education or higher than it was among those with lower education. There was also evidence that the protective association between income and physical IPV was weaker among women who believed that the man should be the primary provider for the family. We acknowledge that the precision of estimates in the models with interaction was low, limiting the inferences we can make regarding interactions.

#### Past year sexual IPV (Tables 2 and 4)

Those in the higher income quartiles at baseline were less likely to have past year experience of sexual IPV than those in the lowest income quartile, though the association was weaker than it was for physical IPV. Furthermore, no longitudinal association was seen between change in income quartile over the course of the study, and odds of sexual IPV at follow-up, in either the intervention or control arm.

**Table 4:** Longitudinal association between woman’s income and past year experience of sexual IPV^a^

	Intervention arm (*n* = 313)			Control arm (*n* = 274)		
	Past year sexual IPV at follow-up n/N (%)	Age-adjusted OR (95%CI)	aOR^b^ (95%CI)	Past year sexual IPV at follow-up n/N (%)	Age-adjusted OR (95%CI)	aOR^b^ (95%CI)
Change in monthly income between baseline and follow-up						
Fallen 1+ quartile/stopped working	21/86 (24%)	–	–	12/77 (16%)	–	–
Stayed in same quartile	16/96 (17%)	0.66 (0.32–1.37)	0.59 (0.27–1.26)	17/78 (22%)	1.57 (0.67–3.72)	1.17 (0.43–3.20)
Increased 1+ quartile/started working	25/112 (22%)	0.95 (0.48–1.86)	1.01 (0.55–1.85)	17/99 (17%)	1.14 (0.46–2.80)	1.00 (0.36–2.80)
Change in financial contribution to household relative to husband between baseline and follow-up						
Was always lower/the same	42/195 (22%)	–	–	22/161 (14%)	–	–
Was higher, now lower/same	6/33 (18%)	0.93 (0.34–2.53)	0.78 (0.31–1.99)	5/30 (17%)	1.72 (0.57–5.22)	1.44 (0.35–5.86)
Was lower/same, now higher	8/45 (18%)	0.81 (0.31–2.07)	0.89 (0.27–2.97)	9/39 (23%)	2.48 (1.01–6.12)	3.21 (1.17–8.84)
Always higher	10/40 (25%)	1.90 (0.89–4.05)	1.98 (0.66–5.97)	16/44 (36%)	6.11 (3.15–11.85)	6.59 (2.54–17.09)

^a^Among women married/living as married at baseline and follow-up

^b^Adjusted for woman’s age, other income/financial contribution variable, partner’s age, baseline measure of outcome, woman’s education, partner’s education, relationship duration, and experience of household-level financial hardship in past year

As for physical IPV, those who contributed more to the household at baseline than their partner did had a somewhat higher risk of sexual IPV (aOR 1.43, 95%CI 0.88–2.34), compared to those contributing the same/less. A similar though stronger association was observed in control women at follow-up, where risk of sexual IPV was higher among women who currently contributed more financially than their husband. Risk was highest among those women who had consistently contributed more throughout the study (baseline and follow-up) (aOR 6.59, 95%CI 2.54–17.09, compared to women who always contributed less/same as partner). This association was not observed among intervention women at follow-up.

With respect to sexual IPV risk, there was no strong evidence of interaction between either economic indicator and the woman’s age, her education, household economic hardship, or her beliefs over whether then man should be the primary provider for the family.

#### Past year economic abuse (Tables 2 and 5)

At baseline, there was no systematic association between a woman’s income quartile and her risk of economic abuse. Neither was there an association between a woman’s changing income over the course of the study and her past year risk of economic abuse at follow-up, in intervention or control communities.

**Table 5:** Longitudinal association between woman’s income and past year experience of economic abuse^a^

	Intervention arm (*n* = 313)			Control arm (*n* = 274)		
	Past year economic abuse at follow-up n/N (%)	Age-adjusted OR (95%CI)	aOR^b^ (95%CI)	Past year economic abuse at follow-up n/N (%)	Age-adjusted OR (95%CI)	aOR^b^ (95%CI)
Change in monthly income between baseline and follow-up						
Fallen 1+ quartile/stopped working	38/86 (44%)	–	–	33/77 (43%)	–	–
Stayed in same quartile	43/96 (45%)	1.00 (0.58–1.72)	1.04 (0.55–1.98)	32/78 (41%)	0.91 (0.54–1.54)	0.82 (0.42–1.62)
Increased 1+ quartile/started working	44/112 (39%)	0.79 (0.51–1.23)	0.84 (0.54–1.29)	36/99 (36%)	0.78 (0.42–1.47)	0.60 (0.31–1.16)

^a^Among women married/living as married at baseline and follow-up

^b^Adjusted for woman’s age, partner’s age, baseline measure of outcome, woman’s education, partner’s education, relationship duration, and experience of household-level financial hardship in past year

There was no strong evidence of interaction between a woman’s income and her age, her education, household economic hardship, or her beliefs over whether then man should be the primary provider for the family.

### Potential pathways between a woman’s income/relative financial contribution and her risk of physical IPV, sexual IPV and economic abuse?

#### Associations between woman’s income quartile and potential pathway variables at baseline (Additional file 4a)

Higher monthly income was associated with decreased household hardship in the past year (among women working), though those not earning anything experienced levels of hardship similar to those in the upper income quartiles.

Those with an income above the first quartile argued less with their partner over his inability/unwillingness to provide for the family, than those in the first quartile. However, these arguments occurred least among those not earning an income at all. There was no clear trend between income and frequent arguments over money/division of resources within the family or accusations that the woman was not fulfilling her responsibilities as wife/mother.

Those in the higher income quartiles were more likely than those in the lowest quartiles to report better communication with their partners, confidence to assert an opinion different to their partner’s, and their partner regularly asking them advice or making them feel appreciated. Those not earning were similar to those in the first quartile with respect to communication, confidence and being asked advice by a partner, but reported similar levels of being made to feel appreciated as those in the higher income quartiles.

Those in the higher income quartiles were more likely than those in the lowest income quartile (or those not earning) to separate from their partner during the course of the study.

#### Associations between woman’s relative financial contribution to household and potential pathway variables at baseline (Additional file 4b)

Women whose financial contribution to the household exceeded that of their partner were more likely to argue frequently about his unwillingness/inability to provide for the family, and about other issues around money/division of resources within the family. The association was in a similar direction though much less strong for accusations that she was not fulfilling her responsibilities as a wife/mother.

Higher relative contributions were also associated with worse communication with the partner, and lower frequency of being asked advice or made to feel appreciated by the partner.

Women who contributed more at baseline were more likely to have separated during the course of the study compared to women who contributed the same/less at baseline.

#### Pathway variables and physical IPV, sexual IPV, economic abuse (Additional file 5)

As hypothesised, household hardship and frequent arguing with the partner over money/gender roles was associated with higher past year physical IPV, sexual IPV and economic abuse. Good communication and relationship dynamics with the partner were associated with lower past year IPV and economic abuse, as was having separated during the course of the study.

## Discussion

We examined two related but distinct aspects of a woman’s economic situation, and explored how they were associated with physical IPV, sexual IPV, and economic abuse, an understudied but pervasive form of partner abuse. While higher income was associated with decreased risk of physical IPV and, to a lesser extent, sexual IPV, women who contributed more financially to the household than their partner did were at increased risk of both physical and sexual IPV. Women’s risk of economic abuse by a partner did not appear to be related to income levels in this sample. This analysis thereby adds to our understanding of how women’s economic empowerment (and interventions to prevent IPV) may affect different forms of abuse in myriad (sometimes adverse) ways. Furthermore, we examined data on a broad range of contextual factors and factors potentially on the pathway between a woman’s income and experiences of partner abuse. Higher income was associated with reduced household hardship, fewer arguments over the partner’s inability to provide for the family, improved relationship dynamics, and increased likelihood of relationship dissolution. Those contributing more than their partner tended to come from more disadvantaged households, argue more over their partner’s inability to provide, and have worse relationship dynamics.

While other studies have demonstrated strong associations between low household income or low socioeconomic status and IPV [43, 51], and many have explored links between women’s employment and IPV, few provide empirical data on associations between women’s income specifically and risk of IPV [52, 53]. In our sample, women’s increasing income was associated with reduced risk of past year IPV, in particular physical IPV, an association that persisted after controlling for the woman’s education, partner’s education and other potential confounders. As hypothesised, our data showed increasing income to be related to less frequent arguing over the man’s failure to fulfil his role as provider and money issues, improved communication and relationship dynamics, and increased confidence, all of which were linked to reduced risk of physical IPV. It was also related to increased levels of separation, again linked to lower IPV risk. While we cannot assess the direction of these associations, they are all potential pathways between income and reduced physical IPV risk, lending support to theories that stress the roles of women’s increased bargaining power [11, 12] and reduced relationship conflict over money and resources [10, 54] in reducing IPV risk. Interestingly, in our sample, higher income was not associated with more frequent accusations that the woman was not fulfilling her role as wife and mother, a potential mechanism through which higher income could lead to greater risk of IPV.

It is also interesting to note that while having a higher income was associated with reduced physical IPV risk compared to being in the lowest income brackets, so too was not earning an income at all. While these results should be interpreted with caution as the number of women not working was small, these women tended to experience lower levels of household hardship and argue less about the man not fulfilling his role as provider than women in the lower income quartiles, suggesting they may have come from more financially secure households. Nevertheless, women not earning an income had worse communication and less confidence to assert an opinion with their partner than women in higher income quartiles, and were not as likely to have separated from their partner during the study, suggesting that participation in work empowers women over and above the financial benefits incurred [7]. The fact that the relationship between income and IPV is n-shaped in this study, with lowest risk observed amongst women at either end of the income extremes (not earning *and* highest income quartiles), cautions against simple binary comparisons of IPV risk between women who are employed and women who aren’t – such analyses may produce null findings just because the measure fails to differentiate the relevant risk categories.

The relationship between higher income and sexual IPV was more complex, only evident in the cross-sectional analysis of baseline data. Changing income over the course of the study was not associated with sexual IPV risk at follow-up. It is plausible that sexual IPV is less heavily influenced by situational triggers (such as depression or arguments within the household) that can result from financial stresses or be mitigated by women’s increasing income. Indeed, recent IPV research has shown differing risk profiles for physical and sexual IPV, with sexual IPV linked most strongly to norms of masculinity that emphasize sexual dominance over women, sexual entitlement within marriage, and toughness and dominance over other men [55]. The fact that prevention interventions (including MAISHA) sometimes have more success at reducing physical IPV than sexual IPV [56–58] is also supportive of the idea that risk factors for physical IPV may be more readily manipulated by, for example, women’s economic participation and social empowerment.

We found no clear association between a woman’s income quartile and past year economic abuse. To date, few studies have explored the relationship between women’s economic participation and economic abuse [59], though concerns have been raised that economic abuse may increase as a result of economic empowerment interventions aimed at the prevention of other forms of IPV. While further research is needed to better understand the relationship between income and economic abuse, our results act as a useful reminder to those working in the field of IPV/abuse prevention, that different forms of abuse may require different types of prevention strategies.

Our findings of elevated physical and sexual IPV risk among women who contribute more financially to the household than their partners do, are consistent with the results of some other studies which have examined IPV in relation to women’s household contributions and situations where the woman works but her husband does not [15, 43, 60]. Relative resource theory emphasises the active threat that a woman earning may pose to the male partner’s status as provider – focusing on the perceived transgression of gender roles that her economic participation may constitute [44, 45]. However, our results (especially when interpreted alongside the results for woman’s income quartile) suggest that the association between relative financial contribution and IPV observed in this study may be as much related to tensions surrounding poverty and/or the partner’s failure to fulfil the expected male role of provider, as to the woman’s economic position (and perceived transgression) per se. The fact that the woman’s higher relative contribution is associated with higher levels of household economic hardship suggests that her higher relative contribution may often reflect the male partner’s low earnings/contributions rather than her high earnings. We also observed that women’s higher relative financial contribution is more strongly related to frequent arguments about the male partner’s inability to provide than it is to arguments about the woman not fulfilling her duties as a wife/mother. Similar tensions also likely underlie the poorer communication within the relationship that is observed where the woman contributes more than her partner, and the increased likelihood of separation. Gender role strain theory posits that men who believe they are failing to fulfil the role of provider may consequently experience negative psychological symptoms and exhibit more aggression towards their female partners [61]. Such patterns have been observed in other qualitative research from Tanzania. For example, in interviews conducted among male informal sector workers in Dar es Salaam and Mbeya, men described how their masculinity and pride could be threatened in situations where their wives were dissatisfied with their low financial contributions to the family, and that this sometimes led to them using violence to maintain respect [62].

As well as a potential causal relationship between relative financial contribution and IPV, it is likely that the two share common risk factors. Many household- and individual-level differences were observed between women who contributed more than their partners and those who contributed the same/less – for example greater household economic hardship and having a partner who is often drunk - which could explain the increased IPV risk experienced by women contributing more. Though we have controlled for several key contextual variables, there may be residual confounding by other unmeasured factors.

Interestingly, change in the woman’s relative financial contribution over the course of the study was related to follow-up risk of IPV in the control arm only. The absence of an association in intervention communities at follow-up suggests that the intervention may have modified the risk association. It is plausible that an intervention targeting gender attitudes would help to lessen the tensions produced by the woman contributing more than the man – not just empowering women to recognise (and be accepted in) their economic role, but also providing space to critique gender norms/expectations around the man’s role as provider. Indeed, an examination of the data (not shown) suggests that women in intervention groups were slightly less likely than women in control groups to believe that a man must be the primary provider for the family and reported fewer arguments about their partner’s inability to provide (though not about the woman’s failure to fulfil her role as wife and mother). They also reported greater levels of confidence to assert an opinion different to their partner’s. These are all potential explanations for how the MAISHA intervention worked to reduce physical IPV (as reported in the primary analysis of intervention impact) [56]. No intervention/control differences were seen regarding communication within the relationship or frequency of separation.

The longitudinal analysis also suggests that control women whose relative financial contribution to the household had been persistently higher than their partner’s (at baseline and follow-up) had even higher risk of IPV than women who only began contributing more during the course of the study (contributed more at follow-up but not at baseline). It has been hypothesised that new threats to the status quo, for example a woman first starting work, might initially increase IPV risk, but as a new ‘normal’ was established that risk would then stabilise or decrease [7]. Our results point more to a picture of cumulative risk. Again, this pattern is perhaps more in keeping with it being the male partner’s poor economic performance or failure to meet gender role expectations that underlies the observed association – with persistent deprivation incurring more risk than short-term disadvantage.

This study has many strengths. Response and retention rates were high across both intervention and control communities. While underreporting of IPV is a concern in such studies, measurement bias was minimised through the use of standardised widely-used questions to measure IPV, administered by interviewers who had received extensive training on conducting surveys relating to IPV. Two rounds of data collection have allowed us to examine longitudinal associations between changing economic circumstances and IPV/abuse risk as well as cross-sectional associations. Furthermore, having data from a CRT has enabled us to compare income/IPV associations between intervention and control arms, allowing insights into how IPV prevention interventions may modify these risk associations.

The study also has a number of limitations. Self-reported income is prone to measurement bias, especially in settings such as this where income often comes from casual or seasonal employment, multiple jobs, and home enterprises, and can vary substantially between seasons or years [63]. Furthermore, income in the form of goods may not be captured by our measure. It is likely that this misclassification would be non-differential with respect to the outcomes of interest, thereby causing us to underestimate the association between income and abuse. Our measure of relative financial contribution to the household also relies on subjective self-reports. While it is entirely plausible that a woman’s experiences of abuse may affect her perception and reporting of relative financial contributions, it is difficult to discern in which direction the bias would operate. Women experiencing abuse could be predisposed to report other aspects of their partner in a negative light, for example downplaying his financial contribution to the household, thereby causing us to overestimate the association between her higher contribution and risk of IPV. However, it is also possible that women who are abused by a partner might underreport their own financial role in the household due to an erosion of self-esteem and confidence, thereby causing us to underestimate the association.

With cross-sectional data it is not possible to establish the direction of an association. We have supplemented our cross-sectional analysis with ‘longitudinal’ data – exploring how change in income/relative financial contribution is related to risk of IPV at follow-up. It is compelling that several associations remain even after controlling for baseline IPV measures, but with only two data-points we are unable to rule out the possibility that the IPV occurred before the change in income/relative financial contribution. Indeed it is likely that the association between income and IPV is bidirectional, with IPV supressing earnings [64] *and* higher income decreasing IPV risk. Similarly, disparity in financial contributions could lead to increased IPV risk, *and* abusive men might be more likely to withhold their own earnings from the household. There is a need for more longitudinal research with multiple rounds of data collection to help further unpick the direction and causal mechanisms of the income/IPV association.

Another limitation is our lack of data on the male partner’s income. While women were asked how much their partner earned on a typical month/week, most did not provide a response to this question (which anyway would likely yield responses with very low validity). Having such data would have allowed us to further explore our hypothesis that the male partner’s low income is an important driver of the relationship between IPV/abuse and the woman’s higher financial contribution to the household relative to her partner’s. The need for dyadic data (data from both members of a couple) to help explore the complex interplay of such factors within a relationship, is increasingly recognised within the field of IPV research.

We recognise that the relationship between income and IPV is context dependent, and that the results of this analysis are not generalizable to other settings, nor necessarily to women in the study area not enrolled in microfinance programmes. Further research from other settings is needed to more fully explore some of the themes that have emerged from this analysis. We have also focused on just two aspects of a woman’s economic situation. Other indicators such as women’s land and property rights/ownership may also be important determinants of IPV risk, and should be considered by future research.

## Conclusions

The different patterns of IPV risk that we observe in relation to a woman’s income and her financial contributions to the household relative to her partner’s, point to the need to take account of a range of household-, relationship-, and partner-level factors when exploring how women’s economic empowerment may impact on IPV risk. We also show that physical IPV, sexual IPV and economic abuse are not related to income in the same way, suggesting that economic interventions to reduce IPV may benefit from complementary components to address economic abuse and sexual IPV. Importantly, our findings suggest that in order to empower women, we need to think beyond broadening women’s access to economic resources and opportunities, and challenging the norms that limit women’s economic participation. Interventions to empower women must also work with both women and men, within couples and at the community-level, to address men’s livelihoods, male gender roles and masculinity norms.

## Additional files

Additional file 1: MAISHA baseline questionnaire. MAISHA baseline survey interview (administered prior to intervention implementation). (DOCX 182 kb)

Additional file 2: MAISHA follow-up questionnaire. MAISHA follow-up survey interview (administered 29 months post baseline). (PDF 529 kb)

Additional file 3: Exposure, outcome, contextual and pathway variables. Details of question items used to construct all exposure, outcome, contextual and pathway variables, and how each variable was constructed and coded. (DOCX 17 kb)

Additional files 4: a Percentage of women in each income category who experienced ‘pathway’ factors in past year (baseline). b Percentage of women in each relative financial contribution category who experienced ‘pathway’ factors in past year (baseline). Tables of baseline associations between income indicators and potential ‘pathway’ factors (DOCX 15 kb)

Additional file 5: Forest plots of odds ratios of association between pathway variables and past year physical IPV, sexual IPV and economic abuse (DOCX 30 kb)

## Data Availability

Datasets analysed in this paper are part of a large MAISHA study involving a second CRT. Because the study is still ongoing, datasets are not publicly available at this point. At the conclusion of our main study analyses, data will be made available upon request and in accordance with NIMR and LSHTM regulations.

## Abbreviations

aOR: adjusted odds ratio
BRAC: Bangladesh Rural Advancement Committee
CI: Confidence interval
CRT: Cluster randomised trial
IPV: Intimate partner violence
OR: Odds ratio
WHO: World Health Organization
