# Supplementary material for: Women’s income and risk of intimate partner violence: secondary findings from the MAISHA cluster randomised trial in North-Western Tanzania
Source: BMC Public Health. 2019 Aug 14;19:1108. doi: 10.1186/s12889-019-7454-1 (PMC6694529; doi:10.1186/s12889-019-7454-1)
Supplement: Supplementary file 1 — MAISHA baseline questionnaire. MAISHA baseline survey interview (administered prior to intervention implementation). (DOCX 182 kb) [file 12889_2019_7454_MOESM1_ESM.docx]

FEMALE PARTICIPANT QUESTIONNIARE

CONFIDENTIAL UPON COMPLETION

SECTION 1: ABOUT YOUR HOUSHOLD 4

SECTION 2: ABOUT YOU AND YOUR PARTNER 6

SECTION 3: ABOUT YOU AND YOUR INCOME 9

SECTION 4: ABOUT YOU AND YOUR HEALTH 13

SECTION 5: ABOUT ATTITUDES AND SOCIAL NORMS 16

SECTION 6: ABOUT YOUR RELATIONSHIP 18

SECTION 7: ABOUT CHILDHOOD 24

SECTION 8: ABOUT YOUR COMMUNITY 26

SECTION 9: ONLY AT FOLLOW UP 28

| **Introduction**  Hello, my name is _____________________ , I am from the National Institute for Medical Research/Mwanza Interventions Trial Unit. As you know, you have agreed to take part in the study we are currently conducting in Mwanza. As part of this study we would like to ask you a number of questions about yourself, your household, your relationship and your community. Some of the questions are sensitive, but everything that you say will be kept private, and will not be shared with others. I would like to encourage you to be honest, as there are no right or wrong answers. At any point you can stop the interview, or choose to not answer a question. This will not affect your further involvement in the study. If you find anything that we discuss upsetting, and would like to talk to someone afterwards, we can help with this.  The interview will take two hours or more to complete. For this it is best if we are not interrupted. Is this a good place to talk, or should we go somewhere else where we can talk privately?  Do you have any questions? |
| --- |

**BEFORE YOU START**

| **Identification** | |
| --- | --- |
| Branch name |  |
| 1. Branch code | **[ ] [ ] [ ] [ ]** |
| 2. Group code | **[ ] [ ] [ ] [ ]** |
| 3. Participant no | **[ ] [ ] [ ]start with 0** |
| **4. P**articipant’s ID | **A [ ] [ ] [ ] [ ] - [ ] [ ] [ ] [ ] - [ ] [ ] [ ]** |

**! PLEASE FILL IN COMPLETE PARTICIPANT ID AT THE BOTTOM OF EVERY PAGE OF THIS QUESTIONNAIRE !**

| **Interview details – START**  Date of interview: **[ ] [ ] /[ ] [ ] [ ]/ [ ] [ ] [ ] [ ] dd/MMM/yyyy**  Time interview started: **[ ] [ ]: [ ] [ ]**  Name of interviewer: **[ ] [ ] [ ]** |
| --- |

| SECTION 1: ABOUT YOUR HOUSHOLD |
| --- |

I would like to start by asking you a few questions about your household. When talking of your household, I mean the place and the people that you normally share food with, and sleep with under the same roof.

MAKE SURE THAT ALL QUESTIONS APPLY TO HER HOUSEHOLD AND NOT THE PLACE YOU ARE CURRENTLY CONDUCTING THE INTERVEIW.

|  | QUESTIONS | | CODING CATEGORIES | | | | |
| --- | --- | --- | --- | --- | --- | --- | --- |
|  | How many people live in your household and share food with you?  PROBE: Does this include children and infants? Does it include any other people who may not be members of your family, such as domestic servants, lodgers or friends who live with you and share food?  MAKE SURE THAT ALL OF THESE PEOPLE ARE INCLUDED IN THE TOTAL | | TOTAL NUMBER OF PEOPLE IN HOUSEHOLD [ ][ ] | | | |  |
|  | Is the head of the household male or female? | | Male  Female | | | | 1  2 |
|  | How long have you been living continuously in Mwanza? | | Years: [ ][ ] Months: [ ][ ] | | | |  |
|  | Is the house you live in rented, owned by you (either on your own, or with someone else), or owned by someone else in your family? | | Rent  Own themselves  Owned by someone else in family  Owned by someone else other than family member  Owned together with someone | | | | 1  2  3  4  5 |
|  | How many rooms does your household use for sleeping? | | [ ][ ] | | | |  |
|  | What type of toilet is mainly used in your household? | | Bush  Uncovered pit latrine  Covered pit latrine shared  Covered pit latrine private  VIP latrine shared VIP latrine private  Flush toilet shared  Flush toilet private  Other (specify)…………………………. | | | | 1  2  3  4  5  6  7  8  9 |
|  | FOR QUESTIONS 106 TO 108 ANSWER THROUGH OBSERVATION IF POSSIBLE. IF THE INTERVIEW IS TAKING PLACE ELSEWHERE, ASK THE RESPONDENT DIRECTLY.  IF THERE IS MORE THAN ONE HOUSE, REFER TO THE MAIN HOUSE. | |  | | | |  |
|  | What is the major construction material of the roof? | | Thatch, Straw  Mud and poles  Tin  Wood  Iron sheet  Tiles  Cement | | | | 1  2  3  4  5  6  7 |
|  | What is the major construction material of the external wall? | | Thatch, Straw  Mud and poles Timber  Un-burnt bricks  Burnt bricks with mud  Burnt bricks with cement  Cement blocks  Stone | | | | 1  2  3  4  5  6  7  8 |
|  | What is the major construction material of the floor? | | Earth  Earth and cow dung  Cement  Mosaic or tiles  Stone | | | | 1  2  3  4  5 |
|  | What is the main source of lighting? | | Electricity  Paraffin, kerosene or gas lantern  Firewood  Candle  Other (Specify)……….………………………… | | | | 1  2  3  4  5 |
|  | What type of fuel do you primarily use for cooking? | | Firewood  Charcoal  Paraffin/kerosene  Electricity  Gas | | | | 1  2  3  4  5 |
|  | Do **you** or any other household member own any of the following: (READ LIST, CIRCLE ALL THAT APPLY)  RESPONDENT REFERS TO HERSELF ONLY. OTHER HOUSHOLD MEMBER REFERS TO THINGS OWNED BY HUSBAND OR OTHERS TOGETHER | | 1. Respondent | | Other household member | | |
|  |  |  |  |  |  |  |  |
|  |  | | Yes | No | Yes | No | |
| a. | Radio | | 1 | 2 | 1 | 2 | |
| b. | Mobile phone | | 1 | 2 | 1 | 2 | |
| c. | Television | | 1 | 2 | 1 | 2 | |
| d. | Fridge | | 1 | 2 | 1 | 2 | |
|  |  | |  | |  | | |
|  | Do you or any other household member own any of the following: | | 1. Respondent | | Other household member | | |
|  |  | | Yes | No | Yes | No | |
| a. | Bicycle | | 1 | 2 | 1 | 2 | |
| b. | Motorcycle/scooter PikiPiki/Boda Boda | | 1 | 2 | 1 | 2 | |
| c. | Dala Dala | | 1 | 2 | 1 | 2 | |
| d. | Car | | 1 | 2 | 1 | 2 | |
|  |  | |  | |  | | |
|  | Do you or any other household member own any of the following: | | 1. Respondent | | Other household member | | |
|  |  | | Yes | No | Yes | No | |
| a. | Agricultural Land | | 1 | 2 | 1 | 2 | |
| b. | Large Livestock (cows, pigs, horses) | | 1 | 2 | 1 | 2 | |
| c. | Small animals (goats, chickens, etc) | | 1 | 2 | 1 | 2 | |
| d. | A business or shop | | 1 | 2 | 1 | 2 | |

| SECTION 2: ABOUT YOU AND YOUR PARTNER |
| --- |
|  |

I would now like to ask you a few questions about yourself and your partner.

|  | QUESTIONS | CODING CATEGORIES | |
| --- | --- | --- | --- |
|  | How old are you?  GET RESPONDENT TO ESTIMATE IF DON'T KNOW EXACTLY | Years: [ ][ ] | |
|  | What is your date of birth?  (RECORD AS MUCH INFORMATION AS POSSIBLE. CODE 96 IF DAY UNKNOWN, 996 IF MONTH UNKNOWN, AND 9696 IF YEAR UNKNOWN) | DAY [ ][ ]  MONTH (MMM) _ _ _  YEAR [ ][ ] [ ][ ] | |
|  | Are you of Tanzanian nationality? | Tanzanian  Not Tanzanian | 1  2 |
|  | Where did you grow up?  PROBE IF NEEDED: Before age 12, where did you live the longest? | This community/neighbourhood  Another rural area/village in Tanzania  Another town/city in Tanzania  Another country  Don’t know/don’t remember  Refused/no answer | 1  2  3  4  96  99 |
|  | How would you describe your religious orientation? | Muslim  Seventh day Adventist  Tanzanian Assemblies of God  Catholic  Lutheran  Moravian  Pentecostal  African Inland Church  No religion  Other (Specify): . . . . . . . . . . . . . . . . . . . | 1  2  3  4  5  6  7  8  9  10 |
|  | What is your ethnic background? | Msukuma  Mjita  Mzinza  Myiramba  Mkara/Mkerewe  Mhaya  Mjaluo  Mkuria/Mshashi  Mchaga  Mhindi  Mwarabu  Other (Specify):. . . . . . . . . . . . . . . . . . . | 1  2  3  4  5  6  7  8  9  10  11  12 |
|  | What is the highest level of schooling you ever completed? | Never went to school  Primary incomplete  Primary complete  Secondary incomplete  Secondary (Form I-IV)  Secondary (Form V-VI)  College training after primary/secondary school and before university  University | 1  2  3  4  5  6  7  8 |

The next few questions are about your current or most recent partner. Please remember that everything you mention will be kept confidential and not share with anyone from your community.

|  | QUESTIONS | CODING CATEGORIES |  | SKIP TO |
| --- | --- | --- | --- | --- |
|  | Are you married or currently living with a man, as if married? | YES  NO | 1  2 | 211 |
| 1. 228 | Have you ever been married or lived with a man as if married? | YES, divorced/separated  Yes, widowed  NO | 1  2  3 | 210  210 |
|  | Are you or have you ever been in a relationship with a man? | Yes  NO | 1  2 | 223 |
|  | Have you been in a relationship with a man during the past 12 months? | Yes  No | 1  2 |  |
|  | The next questions relate to the man that you consider to be your husband or main partner.    USE **PAST TENSE** IF SHE CURRENTLY IS NOT IN A RELATIONSHIP. |  |  |  |
|  | How long have (had) you been in this relationship?  GET TO ESTIMATE IF DON'T KNOW EXACTLY.CODE 00 IF MONTH UNKNOWN | Years: [ ][ ] Months: [ ][ ] |  |  |
|  | Is (was) your partner married to another woman? | Yes  No  DON’T KNOW | 1  2  3 | 214  214 |
|  | Are (were) you the first, second, ... wife/partner?  CODE 96 IF UNKNOWN | Rank: [ ][ ] |  |  |
|  | How old is (was) your partner?  CODE 96 IF UNKNOWN | Years: [ ][ ] |  |  |
| 1. \ | What is(was) your partner’s religion?  CIRCLE ONE | Muslim  Seventh day Adventist  Tanzanian Assemblies of God  Catholic  Lutheran  Moravian  Pentecostal  African Inland Church  Don’t know  Other (Specify):: . . . . . . . . . . . . . . . . . . .. | 1  2  3  4  5  6  7  8  9  10 |  |
|  | What is (was) your partner’s ethnic background?  CIRCLE ONE | Msukuma  Mjita  Mzinza  Myiramba  Mkara/Mikerewe  Mhaya  Mjaluo  Mkuria/Mshashi  Mchaga  Mhindi  Mwarabu  Don’t know  Other . . . . . . . . . . . . . . . . . . . . . . . . | 1  2  3  4  5  6  7  8  9  10  11  12  13 |  |
|  | What is the highest level of education that he completed?  CIRCLE ONE | Never went to school  Primary incomplete  Primary complete  Secondary incomplete  Secondary (Form I-IV)  Secondary (Form V-VI)  College training after primary/secondary school and before university  University  Don’t know | 1  2  3  4  5  6  7  8  9 |  |
|  | Has your partner worked for money during the past 12 months (either employed or self-employed)? | Yes  No | 1  2 | 223 |
|  | Is this work self-employed or is he employed? | Self employed  Employed | 1  2 |  |
|  | Does your partner usually work throughout the year, or does he work seasonally, or only once in a while? | Throughout the year  Seasonally/Part Of The Year  Once in a while | 1  2  3 |  |
|  | Does your partner have to travel away from home for his work? How long would you say he has been away in the past year (12 months)?:  READ RESPONSE CODES CIRCLE ONE | More than half of the year  About half of the year  Less than half of the year  Not all | 1  2  3  4 |  |
|  | Over a typical month/week in the past year, how much money does your partner earn?  USE 969696 IF DON’T KNOW | Month TSH (estimate):[ ][ ] [ ][ ] [ ][ ]  Week TSH (estimate): [ ][ ] [ ][ ] [ ][ ] |  |  |

I would like to learn a bit more about the children that you are responsible for, including both your own children and other children that live with you, as well as children living elsewhere. Please remember this information is confidential and when we are using initials of your children it is only to make sure which child we are talking about. This cannot be used to identify your children!

USE THIS SECTION TO BUILD A RAPPORT WITH THE INTERVIEWEE.

| CHILDREN THAT RESPONSIBLE FOR  Are there children less than 18 months who you are responsible? Yes 1  No 2 (If No go to Question 300) | | | | | | | | | |
| --- | --- | --- | --- | --- | --- | --- | --- | --- | --- |
| **223-224** | How many children up to the age of 18 are you responsible for including those living in the household and those living elsewhere? | | | | No. living with you  [ ] [ ] | | | No. living elsewhere  [ ] [ ] | |
| 225-230 | I will ask you a little about each child in turn , starting with the eldest child. | START WITH OLDEST.  No of child  (1-6) | ⬇ | ⬇ | ⬇ | ⬇ | ⬇ | | ⬇ |
| a. | What is their initials or nickname? | FILL IN **ALL** INITIALS |  |  |  |  |  | |  |
| b. | IS this a boy or a girl? | Female  Male | 1  2 | 1  2 | 1  2 | 1  2 | 1  2 | | 1  2 |
| c. | What is their date of birth? | Day  Month (MMM)  Year  (USE 96 FOR ALL IF NOT KNOWN) | [ ] [ ]  _ _ _  [ ] [ ] [ ] [ ] | [ ] [ ]  _ _ _  [ ] [ ] [ ] [ ] | [ ] [ ]  _ _ _  [ ] [ ] [ ] [ ] | [ ] [ ]  _ _ _  [ ] [ ] [ ] [ ] | [ ] [ ]  _ _ _  [ ] [ ] [ ]  [ ] | | [ ] [ ]  _ _ _  [ ] [ ] [ ] [ ] |
| d. | How old is he/she? | IF DATE UNKNOWN USE 96.  USE 01 IF <1 YEAR | [ ] [ ] | [ ] [ ] | [ ] [ ] | [ ] [ ] | [ ] [ ] | | [ ] [ ] |
| e. | Is [NAME] living with you | No in another household  No away at school  Yes | 1  2  3 | 1  2  3 | 1  2  3 | 1  2  3 | 1  2  3 | | 1  2  3 |
| f. | Are you the mother? | Yes  No - Other woman | 1  2 | 1  2 | 1  2 | 1  2 | 1  2 | | 1  2 |
| g. | Is the father your current partner, or another man? | Current partner  Previous partner  Other man | 1  2  3 | 1  2  3 | 1  2  3 | 1  2  3 | 1  2  3 | | 1  2  3 |
| h. | Is [NAME] at school? | Never SKIP to J  In the past  Currently | 1  2  3 | 1  2  3 | 1  2  3 | 1  2  3 | 1  2  3 | | 1  2  3 |
| i. | What is the highest grade they reached | Primary  Secondary  Nursery  college | 1  2  3  4 | 1  2  3  4 | 1  2  3  4 | 1  2  3  4 | 1  2  3  4 | | 1  2  3  4 |
| j. | IF NOT ENROLLED IN SCHOOL: What is the main reason why they aren't in school  [NB do not read out answers] | Truancy  Pregnancy  Marriage  School fees  Lack of money  Illness of child  Caring for family members  Failed exams  Has “finished”  Farming/  household chores  Too young  Other | 1  2  3  4  5  6  7  8  9  10  11  12 | 1  2  3  4  5  6  7  8  9  10  11  12 | 1  2  3  4  5  6  7  8  9  10  11  12 | 1  2  3  4  5  6  7  8  9  10  11  12 | 1  2  3  4  5  6  7  8  9  10  11  12 | | 1  2  3  4  5  6  7  8  9  10  11  12 |
| k. | IF HAVE LEFT SCHOOL:, What age did they leave at? |  | [ ] [ ] | [ ] [ ] | [ ] [ ] | [ ] [ ] | [ ] [ ] | | [ ] [ ] |
| l. | IF STILL IN SCHOOL: are they currently repeating year? | Yes  No | 1  2 | 1  2 | 1  2 | 1  2 | 1  2 | | 1  2 |

| SECTION 3: ABOUT YOU AND YOUR INCOME |
| --- |
|  |

I would now like to find out a bit more about the ways that you earn money.

|  | What is the main sources of income for you and your family?  CAN ALLOW MULTIPLE RESPONSES | Yes | No | | | | |  |
| --- | --- | --- | --- | --- | --- | --- | --- | --- |
| **a** | Money from own work | 1 | 2 | | | | |  |
| **b.** | Support from husband/partner | 1 | 2 | | | | |  |
| **c.** | Support from other relatives | 1 | 2 | | | | |  |
| **d.** | Pension | 1 | 2 | | | | |  |
| **e.** | Social services/welfare | 1 | 2 | | | | |  |
| **f.** | Other (specify) | …………………………. |  | | | | |  |
|  |  |  | | | | | |  |
|  | Have you personally earned money during the past 12 months? | Yes  No | | | 1  2 | | | 320 |
|  | Are you self-employed or do you work for someone else/organisation? | Self employed  Worked for someone else or an organization  Both | | | 1  2  3 | | | 310 |
|  | (IF WORKING FOR SOMEONE ELSE OR AN ORGANISATION),  Who is your main employer? | Relative  Neighbor  Friend / acquaintance  Government  NGO  Private company / Someone’s business  Other (Specify):…….. . . . . . . . . . . . . . . . . . . . . . . | | | 1  2  3  4  5  6  7 | | |  |
| The following questions refer only to work you did while employed by someone else or an organization  Skip if not employed by someone else | | | | | | | | |
|  | Is this a regular or an occasional activity? | Regular  Occasional | | | 1  2 | | |  |
|  | Do you usually work throughout the year, or do you work seasonally, or only once in a while? | Throughout the year  Seasonally/Part Of The Year  Once in a while | | | 1  2  3 | | |  |
|  | For how many months in total did you do this activity during the past year? Would you say | 1 month or less  1-6 months  6-9 months  9-12 months | | | 1  2  3  4 | | |  |
|  | Are you paid in cash, given food or other items, or are you not paid at all? | Cash only  Cash and items  Items only  Other  Not paid | | | 1  2  3  4  5 | | | 309  309  309 |
|  | On a typical working day/week/month how much do you earn from this activity?  FILL OUT ONLY ONE, EITHER DAY, WEEK OR MONTH. ENTER 969696 IF DON’T KNOW | Day: [ ] [ ] [ ] [ ] [ ] [ ]  Week: [ ] [ ] [ ] [ ] [ ] [ ]  Month: [ ] [ ] [ ] [ ] [ ] [ ] | | | | |  | |
|  | Do you plan to continue this activity over the next 12 months? | Yes  No  Don’t know | | | 1  2  96 | | |  |
|  | If self-employed… |  | | | | | |  |
|  | What kind of business do you run?  (MULTIPLE RESPONSES OK) | a. Selling vegetables  b. Selling homemade food  c. Running small restaurant  d. Sewing or fixing cloths  e. Selling cloths  f. Brewing  g. Running a salon  h. Running a shop  I. Selling fish  j. Other (Specify):……….……………… | | Yes  1  1  1  1  1  1  1  1  1 | No  2  2  2  2  2  2  2  2  2 |  |  |  |
|  | Have you been operating this business for more than or less than 12 months? | Less than 12 months  More than 12 months | | | 1  2 | | |  |
|  | Are you mainly responsible for this activity, or are others responsible? | I am mainly responsible  Others are responsible | | | 1  2 | | |  |
|  | How many other household members contributed time, money or ideas to this activity in the last year?  ENTER 96 IF DON’T KNOW | Number: [ ] [ ] | | | | | |  |
|  | For how many days did you do this activity during the past month?  ENTER 96 IF DON’T KNOW | Number: [ ] [ ] | | | | | |  |
|  | On a typical working day, how many hours do you work?  ENTER 96 IF DON’T KNOW | Hours: [ ] [ ] | | | | | |  |
|  | On a typical working day/week/month how much do you earn from this activity?  FILL OUT EITHER DAY, WEEK OR MONTH | Day: [ ] [ ] [ ] [ ] [ ] [ ]  Week: [ ] [ ] [ ] [ ] [ ] [ ]  Month: [ ] [ ] [ ] [ ] [ ] [ ] | | | | | |  |
|  | During the past month, how many times did you not earn enough to cover the costs of running your business? | Never  Once  Few times  Many times | | | 1  2  3  4 | | |  |
|  | Do you employ other people and pay them a wage? | Yes  No | | | 1  2 | | |  |
|  | Of the money you earned over the past month, what proportion did you reinvest to maintain or expand your business? | None of it  Some of if  Half of it  Most of it  All of it | | | 1  2  3  4  5 | | |  |

|  | QUESTIONS | CODING CATEGO RIES | | |  | SKIP TO |
| --- | --- | --- | --- | --- | --- | --- |
|  | I would now like to ask you some questions about whether you have been using a microfinance provider, such as SACCOS, BRAC, FINCA or others? | | | | |  |
|  | In the past 12 months,Have you ever borrowed money from a microfinance provider, different from BRAC like SACCOS, BRAC, FINCA or others? | Yes  No | | | 1  2 | 324 |
|  | Who have you borrowed money from any organization different from BRAc in the past 12 months? | a. FINCA  b SACCOS  c. PRIDE  d. JIJENGE  e. Other (specify)………….…  . | Yes  1  1  1  1  1 | No  2  2  2  2  2 | |  |
|  | Are you still borrowing money from one of these organization? | Yes  No | | 1  2 | |  |
|  | About your current loan which was not from BRAC,, how much money did you borrow?  USE 0 IN ALL SPACES TO WRITE IF ONLY BRAC LOAN AND NOT OTHER | Amount [ ] [ ] [ ] [ ] [ ] [ ] [ ] | | |  |  |
|  | Are you currently borrowing money from BRAC? | Yes  No | | | 1  2 | 334 |
|  | When did you join BRAC?  (USE 96 IF NOT KNOWN FOR MONTH, 9696 FOR YEAR) | Month [ ] [ ]  Year [ ] [ ] [ ] [ ] | | |  |  |
|  | When did you receive your first loan from BRAC?  (USE 96 IF NOT KNOWN FOR MONTH, 9696 FOR YEAR) | Month [ ] [ ]  Year [ ] [ ] [ ] [ ] | | |  |  |
|  | About your current loan you got from BRAC, how much money did you borrow? | Amount [ ] [ ] [ ] [ ] [ ] [ ] [ ] | | |  |  |
|  | In the past 12 months, have you been continuously  borrowing money from BRAC, or where there times when you did not borrow money from them? | Continuously  Interrupted  New loan | | | 1  23 | 330  330 |
|  | Why did you interrupt borrowing money from BRAC? | Did not need it any more  Could not pay back the loan  Husband refused to take new loan  Other (specify)………………………………… | | | 1  2  3  4 |  |
|  | How do/did you **primarily** use the money you borrow?  READ OUT RESPONSES | To build a small business  To maintain a business  To pay for medical expenses  To pay for school expenses  To buy food or clothes  To pay back another microfinance loan  To pay for rent  To help other family members  Others (specify) ………………………….. | | | 1  2  3  4  5  6  7  8  9 |  |
|  | How confident do you feel about being able to pay this back? Would you say that you feel:  Very confident  Somewhat confident  Not very confident | Very confident  Somewhat confident  Not very confident | | | 1  2  3 |  |
|  | How important is the money that you get from BRAC and other microfinance groups? Would you say that it is extremely important, somewhat important, or not very important at all. | Extremely important  Somewhat important  Not very important | | | 1  2  3 |  |
|  | In the past 12 months, overall, how has you being a member of BRAC or other microfinance groups influenced your relationship with your partner? Would you say that its has:= had not effect on the relationship, made your relationship more difficult, or improved your relationship? | Not effected the relationship  Made your relationship more difficult  Improved your relationship  Not in a relationship | | | 1  2  3  9 |  |

|  | QUESTIONS | CODING CATEGORIES | | | |
| --- | --- | --- | --- | --- | --- |
|  | Nowadays, many families have a hard time making ends meets. I would like to learn more about how your household is coping. During the last 12 months, how many times…  EMPHASIZE THAT YOU ARE TALKING ABOUT THE PAST **12 MONTHS**  **In the past 12 months…….** |  | If **not** **NEVER**… | | |
|  |  | Never | Once | Few times | Many times |
| a. | … were you very worried/stressed about your general financial situation.  Would you say ,this has happened or never happened? | 1 | 2 | 3 | 4 |
| b. | … have you had trouble buying food or other necessities for your family?  Would you say ,this has happened or never happened? | 1 | 2 | 3 | 4 |
| c. | … have you had to borrow money to pay rent or other bills ?  Would you say ,this has happened or never happened? | 1 | 2 | 3 | 4 |
| d. | … did any of your family members need to see a doctor but could not because you did not have enough money?  Would you say ,this has happened or never happened? | 1 | 2 | 3 | 4 |
| e. | … did your children miss days of school because you did not have money for school fees, uniforms or supplies?  Would you say ,this has happened or never happened? | 1 | 2 | 3 | 4 |
| f. | … have you or any of your own children gone a whole day without eating anything because there was not enough food?  Would you say ,this has happened or never happened? | 1 | 2 | 3 | 4 |

|  | QUESTIONS | CODING CATEGORIES | | |
| --- | --- | --- | --- | --- |
|  | During the past 12 months, how important is the money that you yourself bring into the family? Would you say that it is extremely important, somewhat important, or not very important at all. | | Extremely important  Very important  Somewhat important  Not very important  Don’t bring in any money | 1  2  3  4  5 |
|  | Would you say that the money that you bring into the household is more than what your husband/partner contributes, less than what he contributes, or about the same as he contributes? | | Less than partner  Same as Partner  More than partner  Has no partner | 1  2  3  4 |
|  | Do you think that you would be able to look after yourself and your family on your income alone? Would you say Definitely yes, yes with difficulty, probably not, or definitely not. | | Yes definitely  Yes with difficulty  Probably not  Definitely not | 1  2  3  4 |

| SECTION 4: ABOUT YOU AND YOUR HEALTH |
| --- |
|  |

|  | I would like to learn about your approach to life, in general. I am going to read you some statements, and I would like you to say whether they are true or false:  If true,  Ask somewhat true, true or definitely true  If not true,  Ask, somewhat false, false, or definitely false | Definitely true | Mostly true | Slightly true | Slightly false | Mostly false | Definitely f false |
| --- | --- | --- | --- | --- | --- | --- | --- |
| a. | You can think of many ways to get out of a difficult situation when life evolves unexpectedly. | 1 | 2 | 3 | 4 | 5 | 6 |
| b. | You energetically pursue your goals. | 1 | 2 | 3 | 4 | 5 | 6 |
| c. | You feel tired most of the time. | 1 | 2 | 3 | 4 | 5 | 6 |
| d. | There are lots of ways around any problem. | 1 | 2 | 3 | 4 | 5 | 6 |
| e. | You are easily downed in an argument. | 1 | 2 | 3 | 4 | 5 | 6 |
| f. | You can think of many ways to get the things in life that are important to you. | 1 | 2 | 3 | 4 | 5 | 6 |
| g. | You worry about your health. | 1 | 2 | 3 | 4 | 5 | 6 |
| h. | Even when others get discouraged, you know you can find a way to solve the problem. | 1 | 2 | 3 | 4 | 5 | 6 |
| i. | Your past experiences have prepared you well for your future. | 1 | 2 | 3 | 4 | 5 | 6 |
| j. | You ’ve been pretty successful in life. | 1 | 2 | 3 | 4 | 5 | 6 |
| k. | You usually find yourself worrying about something. | 1 | 2 | 3 | 4 | 5 | 6 |
| l. | You meet the goals that you set for myself. | 1 | 2 | 3 | 4 | 5 | 6 |

I would now like to ask you some questions about your health.

|  |  |  | |
| --- | --- | --- | --- |
|  | During the last 4 weeks, have you been bothered by any of the following problems? | Yes | No |
| a. | Do you often have headaches? | 1 | 2 |
| b. | Is your appetite poor? | 1 | 2 |
| c. | Do you sleep badly? Like difficulties falling asleep, waking up in the middle of the night more than 3 times or waking up early in the morning and not getting back to sleep. | 1 | 2 |
| d. | Are you easily frightened? | 1 | 2 |
| e. | Do your hands shake? | 1 | 2 |
| f. | Do you feel nervous, tense or worried? | 1 | 2 |
| g. | Is your digestion poor? Like you are often constipated, feel nauseous, or you don’t have an appetite. | 1 | 2 |
| h. | Do you have trouble thinking clearly? | 1 | 2 |
| i. | Do you feel unhappy? | 1 | 2 |
| j. | Do you cry more than usual? Like every day or more than once per day, because of problems? | 1 | 2 |
| k. | Do you find it difficult to enjoy your daily activities? | 1 | 2 |
| l. | Do you find it difficult to make decisions? | 1 | 2 |
| m. | Is your daily work suffering? | 1 | 2 |
| n. | Are you unable to play a useful part in life? | 1 | 2 |
| o. | Have you lost interest in things? | 1 | 2 |
| p. | Do you feel that you are a worthless person? | 1 | 2 |
| q. | Has the thought of ending your life been on your mind? | 1 | 2 |
| r. | Do you have uncomfortable feelings in your stomach? | 1 | 2 |
| s. | Are you easily tired? | 1 | 2 |

I would now like to ask you some questions about your reproductive health. Some of the questions may be embarrassing to answer. Please remember that this information is confidential and you may choose not to answer them.

| 402 | Have you ever been pregnant?  IF YES: How many times? | Number [ ][ ]  99 if Never | | | | |  | | 405 |  |  |
| --- | --- | --- | --- | --- | --- | --- | --- | --- | --- | --- | --- |
|  | How many children have you given birth to? | Number | | | | | [ ][ ] | |  |  |  |
|  | How many of these children are alive?  CODE 0 IF NOT ALIVE | Number | | | | | [ ][ ] | |  |  |  |
|  | Are you currently using modern contraceptive methods like hormonal contraceptives, loop or alike? | Yes  No  Pregnant | | | | | 1  2  3 | |  |  |  |
|  | At what age did you first have sexual intercourse? | Age in years: [ ][ ]  99 if Never had sex | | | | |  | | 411 |  |  |
|  | How many people in total have you had sexual intercourse with in your life? | Give total number: | | | | | [ ][ ] | |  |  |  |
|  | How many people in total have you had sexual intercourse with in the past year?  CODE 99 IF HAS REFUSED TO ANSWER.  IF 00 SKIP TO QN 411 | Give total number: | | | | | [ ][ ] | |  | |  |
|  | During the last 12 months, have you ever received money or material goods in exchange for sex? | Yes  No  No response | | | | | 1  2  99 | |  | |  |
|  | In the past 12 months, when you had sex, how often did you use a condom? Would you say:  READ RESPONSES | Almost always  Most of the time  Some times  Almost never | | | | | 1  2  3  4 | |  | |  |
|  | I don’t want to know the result, but in the past year, *have you* had an HIV test? | Yes  No  No response | | | | | 1  2  99 | |  | |  |
| Increasingly women and men in Tanzania are drinking alcoholic beverages. If you don't mind, I would like to ask you about yours and your husband/partner’s use of alcohol. Please feel free to be open. | | | | | | | | | | | |
|  | In the past 12 months, have you ever drunk an alcohol-containing beverage? For example beer, wine, local brew, local spirit (eg. Gongo) or other alcoholic beverage? | Yes  No | | | | | 1  2 | | 416 | |  |
|  | How often do you have a drink containing alcohol? Would you say:  READ RESPONSES: | 1 -6 times per year  2-4 times a month  2-3 times a week  4 or more times a week | | | | | 1  2  3  4 | |  | |  |
|  | On average, how many drinks containing alcohol do you have on a typical day when you are drinking? | 1 or 2  3 or 4  5 or 6  7, 8 or 9  10 or more | | | | | 1  2  3  4  5 | |  | |  |
|  |  | | No | Once per year | Once per every month | Once per every week | | Daily, almost daily |  | | |
| a. | Do you use six or more drinks on one occasion?  IF YES , READ OUT ANSWERS | | 1 | 2 | 3 | 4 | | 5 |  | | |
| b. | In the past 12 months, have you found that you were not able to stop drinking once you had started?  IF YES , READ OUT ANSWERS | | 1 | 2 | 3 | 4 | | 5 |  | | |
| c. | In the past 12 months ,have you failed to do what was normally expected of you because of drinking?  IF YES , READ OUT ANSWERS | | 1 | 2 | 3 | 4 | | 5 |  | | |
| d. | In the past 12 months, have have you needed a first drink in the morning to get yourself going after a heavy drinking session?  IF YES , READ OUT ANSWERS | | 1 | 2 | 3 | 4 | | 5 |  | | |
| e. | In the past 12 months, have you had a feeling of guilt or remorse after drinking?  IF YES , READ OUT ANSWERS | | 1 | 2 | 3 | 4 | | 5 |  | | |
| f. | In the past 12 months, have you been unable to remember what happened the night before because of your drinking?  IF YES , READ OUT ANSWERS | | 1 | 2 | 3 | 4 | | 5 |  | | |
| g. | Have you or someone else been injured because of your drinking – either in the past 12 months, or before this? | | Yes, during the past 12 months  Yes, but not in the last year  No | | | | | 1  2  3 |  | | |
| h. | Has a relative, friend, doctor, or other health care worker been concerned about your drinking or suggested you cut down? | | Yes, during the past 12 months  Yes, but not in the last year  No | | | | | 1  2  3 |  | | |
|  | Does your partner drink alcohol? | | Yes  No  No partner | | | | | 1  2  3 | 418  Section 5 | | |
|  | In the past 12 months, how often have you seen your partner intoxicated (drunk)? | | Never  Once  Few times  Many times | | | | | 1  2  3  4 |  | | |
|  | In the past 12 months, how often have you seen your partner physically fight with other people you are not living with? | | Never  Once  Few times  Many times | | | | | 1  2  3  4 |  | | |

| SECTION 5: ABOUT ATTITUDES AND SOCIAL NORMS |
| --- |
|  |

In this community and elsewhere, people have different ideas about families and what is acceptable behavior for men and women in the home. We would like to know your views on what is acceptable and what you think other people in your community believe.

|  | QUESTIONS | CODING CATEGORIES | | | | | | | |
| --- | --- | --- | --- | --- | --- | --- | --- | --- | --- |
| 500-501 | I am going to make a number of statements about men and women in general. When I read the following statements can you please indicate how much you personally agree or disagree ,Then, please indicate how much you think your friends and neighbors would agree or disagree with the same statements.  READ STATEMENTS, THEN ASK IF AGREE OR DISAGREE.  THEN ASK IF AGREE OR STRONGLY AGREE OR DISAGREE OR STRONGLY DISAGREE | In your personal opinion… | | | | 1. Thinking now about your friends and neighbours, would you say that the majority of them would... | | | |
|  |  | I strongly agree | I agree | I disagree | I strongly disagree | They strongly agree | They agree | They disagree | They strongly disagree |
|  | A couple should decide together things that affect the health and well-being of the family | 1 | 2 | 3 | 4 | 1 | 2 | 3 | 4 |
|  | It’s a wife’s obligation to have sex with her husband even if she doesn’t want to | 1 | 2 | 3 | 4 | 1 | 2 | 3 | 4 |
|  | It **must** be the man who is the primary provider for the family. | 1 | 2 | 3 | 4 | 1 | 2 | 3 | 4 |
|  | Women should have the same right as men to study and to work outside of the home | 1 | 2 | 3 | 4 | 1 | 2 | 3 | 4 |
|  | A woman should obey her husband’s wishes even if she disagrees. | 1 | 2 | 3 | 4 | 1 | 2 | 3 | 4 |
|  | Even healthy relationships can include hitting each other as long as the partners love each other. | 1 | 2 | 3 | 4 | 1 | 2 | 3 | 4 |
|  | It is perfectly acceptable for women to work outside the home to help support the family economically | 1 | 2 | 3 | 4 | 1 | 2 | 3 | 4 |
|  | The leadership of a community should be largely in the hands of men. | 1 | 2 | 3 | 4 | 1 | 2 | 3 | 4 |
|  | Children and men would benefit, if fathers were more involved in caring for their children | 1 | 2 | 3 | 4 | 1 | 2 | 3 | 4 |
|  | Sons in the family should be given more encouragement to go to school than daughters | 1 | 2 | 3 | 4 | 1 | 2 | 3 | 4 |
|  | It is natural and right that men have more power than woman in the family | 1 | 2 | 3 | 4 | 1 | 2 | 3 | 4 |
|  | Women could take on many of the roles of men, if men were willing to share power. | 1 | 2 | 3 | 4 | 1 | 2 | 3 | 4 |

| 502-503 | People have different opinions about whether there are situations where a man can be violent towards his partner. When I read the following statements can you please say whether you personally AGREE OR DISAGREE, Then, please can you say whether you think that your friends ,neighbors, wazazi would agree or disagree.  PROBE WHERE NEEDED.  READ SENTENCE AND ASK IF AGREE , OR STRONGLY AGREE,  THEN, ASK IF DISAGREE or STRONGLY DISAGREE | 1. In my personal opinion… | | | | 1. Thinking now about your friends and neighbours, would you say that the majority of them would... | | | |  |
| --- | --- | --- | --- | --- | --- | --- | --- | --- | --- | --- |
|  |  | I strongly agree | I agree | I disagree | I strongly disagree | They strongly agree | They agree | They disagree | They strongly disagree |  |
|  | A man has a good reason to hit his wife if **she** does not complete her household work to his satisfaction | 1 | 2 | 3 | 4 | 1 | 2 | 3 | 4 |  |
|  | A man has a good reason to hit his wife if **she** disobeys him | 1 | 2 | 3 | 4 | 1 | 2 | 3 | 4 |  |
|  | A man have good reason to hit his wife if **she** refuses to have sexual intercourse with him |  |  |  |  |  |  |  |  |  |
|  | A man does not have any reason to hit his wife in any way | 1 | 2 | 3 | 4 | 1 | 2 | 3 | 4 |  |
|  | A man has a good reason to hit his wife if **she** protests because he has other girlfriends | 1 | 2 | 3 | 4 | 1 | 2 | 3 | 4 |  |
|  | A man has a good reason to hit his wife if **he** suspects that she is unfaithful in marriage | 1 | 2 | 3 | 4 | 1 | 2 | 3 | 4 |  |
|  | A man has a good reason to hit his wife if he finds out that she has been unfaithful in marriage | 1 | 2 | 3 | 4 | 1 | 2 | 3 | 4 |  |
|  | A woman should tolerate violence in order to keep her family together | 1 | 2 | 3 | 4 | 1 | 2 | 3 | 4 |  |
|  | Violence between husband and wife is a private matter and others should not intervene | 1 | 2 | 3 | 4 | 1 | 2 | 3 | 4 |  |
|  | People should do everything they can to support a woman who has left an abusive husband | 1 | 2 | 3 | 4 | 1 | 2 | 3 | 4 |  |
|  | A woman who is raped should not tell anyone, or she will be blamed for the attack | 1 | 2 | 3 | 4 | 1 | 2 | 3 | 4 |  |
|  | People have a duty to intervene if they hear or see a woman being hit | 1 | 2 | 3 | 4 | 1 | 2 | 3 | 4 |  |
|  | A woman who abandons her abusive husband brings shame upon her family | 1 | 2 | 3 | 4 | 1 | 2 | 3 | 4 |  |

|  | Now I would like to ask you about your opinion of certain types of behaviours. Please tell me whether or not you consider the following actions a form of violence in all cases, in some cases, or never. Please feel free to speak honestly.  Would you say that it is violence if: | YES | In some cases | NO |
| --- | --- | --- | --- | --- |
| a. | A parent slaps a child for disrespecting his elders | 1 | 2 | 3 |
| b. | A man hits his wife because she has done something wrong, but leaves no bruises/marks | 1 | 2 | 3 |
| c. | A wife repeatedly belittles and humiliates her husband in public | 1 | 2 | 3 |
| d. | A parent yells at a child to stay out of the street | 1 | 2 | 3 |
| e. | A woman refuses to have sex with her husband for a week | 1 | 2 | 3 |
| f. | A man hits a woman during an argument but then apologizes | 1 | 2 | 3 |
| g. | A girl gets a boy sexually aroused but does not allow him to go further | 1 | 2 | 3 |
| h. | A man refuses to give his wife money to sustain the family even when he has money for other things | 1 | 2 | 3 |
| i. | A man threatens to hit a woman but does not actually hit her | 1 | 2 | 3 |
| j. | A man forces a woman he does not know to have sex | 1 | 2 | 3 |
| k. | A man provides for his family but keeps part of his earnings for himself | 1 | 2 | 3 |
| l. | A husband forces his wife have sex even though she does not want to | 1 | 2 | 3 |

| SECTION 6: ABOUT YOUR RELATIONSHIP |
| --- |

**PLEASE LOOK AT QUESTION 209. IF THE RESPONSE IS NO GO TO SECTION 7. IF QUESTION 210 IS NO PARTNER IN PAST 12 MONTHS, START AT 607.**

When two people marry, live together or are in a relationship, they usually share both good and bad moments. I would now like to ask you some question about your current and past relationships and how your husband/partner treats (treated) you. If anyone interrupts us I will change the topic of conversation. I would again like to assure you that your answers will be kept secret, and that you do not have to answer any question that you do not want to. May I continue?

|  | QUESTIONS | CODING CATEGORIES | | | | | |
| --- | --- | --- | --- | --- | --- | --- | --- |
|  | During the last 12 months, did you and your partner discuss the following topics together |  | If happed fill the answer in the shaded area | | | |  |
|  |  | Never | Once | Few times | | Many times |  |
| a. | … things that happened to you during the day? Would you say never, once, a few times, or many times? | 1 | 2 | 3 | | 4 |  |
| b. | … things that happened to him in the day?  Would you say never, once, a few times, or many times? | 1 | 2 | 3 | | 4 |  |
| c. | … your worries or feelings?  Would you say never, once, a few times, or many times? | 1 | 2 | 3 | | 4 |  |
| d. | … his worries or feelings?.  Would you say never, once, a few times, or many times? | 1 | 2 | 3 | | 4 |  |
|  | During the last 12 month, did your partner ever… |  | If yes, how often? | | | |  |
| a. | … ask you for your advice to resolve a problem he was facing?  Would you say never, once, a few times, or many times? | 1 | 2 | 3 | 4 | |  |
| b. | … followed your advice to resolve a problem he was facing?  Would you say never, once, a few times, or many times? | 1 | 2 | 3 | 4 | |  |
| c. | … helped you in finding work?  Would you say never, once, a few times, or many times? | 1 | 2 | 3 | 4 | |  |
| d. | … encouraged you to participate in something outside of the home that was only for *your* benefit?  Would you say never, once, a few times, or many times? | 1 | 2 | 3 | 4 | |  |
| e. | … made you feel appreciated.  Would you say never, once, a few times, or many times? | 1 | 2 | 3 | 4 | |  |
|  |  |  |  |  |  | |  |

|  | How confident do you feel to assert your own opinion if it is different from that of your husband? | Very confident  Confident but would need to be encouraged  Not confident at all  Don’t know | 1  2  3  96 |
| --- | --- | --- | --- |
|  | Have you asserted your opinion in the past 12 months? | No  Yes | 2  1 |
|  | How comfortable would you feel in resisting efforts by your husband to control aspects of your life like who you see and how you spend your money?  READ ANSWERS | Very comfortable  Comfortable but would need encouragement  Not comfortable at all  Don’t know | 1  2  3  96 |

|  | No matter how well a couple gets along, there are times when they disagree. **In your relationship with your current / most recent partner….** |  |  | | |
| --- | --- | --- | --- | --- | --- |
|  |  | Yes | No |  |  |
| a. | … would you say that you quarreled in the past 12 months?  (SKIP TO QUESTION 607 IF ANSWER IS NO) | 1 | 2 |  |  |
|  | How often have you quarreled about:  IF HAPPENNED, HAS HAPPENED ONCE, FEW TIMES OR MANY TIMES? | Never | Once | Few times | Many times |
| b. | Accusations that you are not fulfilling your responsibilities as wife and mother | 1 | 2 | 3 | 4 |
| c. | His inability or unwillingness to provide for the family | 1 | 2 | 3 | 4 |
| d. | Other issues around money and division of resources in the family | 1 | 2 | 3 | 4 |
| e. | His drinking/gambling or drug use | 1 | 2 | 3 | 4 |
| f. | Your drinking | 1 | 2 | 3 | 4 |
| g. | Concerns about outside partners or accusations of infidelity | 1 | 2 | 3 | 4 |
| h. | Your refusal to have sex | 1 | 2 | 3 | 4 |
| i. | Other issues around sex (frequency, condom use, etc) | 1 | 2 | 3 | 4 |
| j. | You disobeying your partner or treating him disrespectfully. | 1 | 2 | 3 | 4 |
| k. | Him treating you or your children disrespectfully. | 1 | 2 | 3 | 4 |
| l. | Him being unhappy that you had taken a loan from a microfinance organization. | 1 | 2 | 3 | 4 |

|  | In the past 12 months when you have argued with your partner, how generally did you react?  Would you say that you did the following activities never, once, a few times or many times: |  | If happed fill the answer in the shaded area | | |
| --- | --- | --- | --- | --- | --- |
|  |  | Never | Once | Few times | Many times |
|  | Expressed how you felt in a calm and respectful way | 1 | 2 | 3 | 4 |
|  | Tried to see your partner’s side and listened carefully to what he had to say. | 1 | 2 | 3 | 4 |
| c. | You brought in or tried to bring in someone to help settle things. | 1 | 2 | 3 | 4 |
| d. | When you felt the argument got too heated, you left so that you and your partner had time to calm down. | 1 | 2 | 3 | 4 |
| e. | You started to make complaints about things unrelated to your initial argument. | 1 | 2 | 3 | 4 |
| f. | You insulted or swore at him. | 1 | 2 | 3 | 4 |
| g. | You yelled | 1 | 2 | 3 | 4 |
| h. | You pushed, shaked or pulled him | 1 | 2 | 3 | 4 |

| 607- 608 | I am now going to ask you about some situations that are true for many women. Thinking about your (current or most recent or past) husband/partner, would you say it is generally true that he: |  | | ONLY ASK IF ‘YES’ IN 607  Has this happened in the past 12 months? | |
| --- | --- | --- | --- | --- | --- |
|  |  | Yes | No | Yes | No |
| a. | Tries to keep you from seeing your friends | 1 | 2 | 1 | 2 |
| b. | Tries to restrict contact with your family of birth | 1 | 2 | 1 | 2 |
| c. | Insists on knowing where you are at all times | 1 | 2 | 1 | 2 |
| d. | Is jealous and gets angry if you speak with another man | 1 | 2 | 1 | 2 |
| e. | Is often suspicious that you are unfaithful | 1 | 2 | 1 | 2 |
| 609 - 610 | Thinking about your (current or most recent/past) husband/partner, would you say it is generally true that he: |  | | ONLY ASK IF ‘YES’ IN 609  Has this happened in the past 12 months? | |
| a. | Refuses to give you enough money for household expenses, even when he has money for other things? | 1 | 2 | 1 | 2 |
| b. | Takes money that you have earned away from you | 1 | 2 | 1 | 2 |
| c. | Makes important financial decisions without consulting you | 1 | 2 | 1 | 2 |

| 611 – 614 | The next questions are about things that happen to many women, and that your current partner, or any other partner may have done to you.  Has your current husband/partner, or any other partner ever…. | (**If YES continue with 612  If NO go to 611 next sub question, if ALL NO skip to 616**)  YES NO | | Has this happened in the past 12 months?  (**If YES ask 613 only. If NO ask 614 only**)  YES NO | In the past 12 months would you say that this has happened once, a few times or many times?  One Few Many | Before the past 12 months would you say that this has happened once, a few times or many times?  One Few Many |
| --- | --- | --- | --- | --- | --- | --- |
| a. | Insulted you or made you feel bad about yourself? | 1 2 | | 1 2 | 1 2 3 | 1 2 3 |
| b. | Belittled or humiliated you in front of other people? | 1 2 | | 1 2 | 1 2 3 | 1 2 3 |
| c. | Done things to scare or intimidate you on purpose (e.g. by the way he looked at you, by yelling and smashing things)? | 1 2 | | 1 2 | 1 2 3 | 1 2 3 |
|  | Verbally threatened to hurt you or someone you care about? | 1 2 | | 1 2 | 1 2 3 | 1 2 3 |
|  | SKIP IF 611 ALL NO  Was the person who insulted, humiliated, scared or threatened you your current or a previous partner? | | Current 1  Previous 2  Both 3 | | | |

| 616 - 619 | Has your current partner or any other partner ever…. | (**If YES continue with 617  If NO go next 616 sub question if ALL NO skip to 621**)  YES NO | | Has this happened in the past 12 months?  (**If YES ask 618 only. If NO ask 619 only**)  YES NO | In the past 12 months would you say that this has happened once, a few times or many times?  One Few Many | Before the past 12 months would you say that this has happened once, a few times or many times?  One Few Many |
| --- | --- | --- | --- | --- | --- | --- |
| a. | Slapped you or thrown something at you that could hurt you? | 1 2 | | 1 2 | 1 2 3 | 1 2 3 |
| b. | Pushed you or shoved you or pulled your hair? | 1 2 | | 1 2 | 1 2 3 | 1 2 3 |
| c. | Hit you with his fist or with something else that could hurt you? | 1 2 | | 1 2 | 1 2 3 | 1 2 3 |
| d. | Kicked you, dragged you or beaten you up? | 1 2 | | 1 2 | 1 2 3 | 1 2 3 |
| e. | Choked or burnt you on purpose? | 1 2 | | 1 2 | 1 2 3 | 1 2 3 |
| f. | Threatened to use or actually used a gun, knife or other weapon against you? | 1 2 | | 1 2 | 1 2 3 | 1 2 3 |
|  | SKIP IF 616 ALL NO  Was the person who did these things to you your current partner, or a previous partner? | | | Current 1  Previous /PAST 2  Both 3 | | |
| 621 - 624 |  | | (**If YES continue with 622.  If NO go to next 621 sub question, if ALL NO skip to 626**)  YES NO | Has this happened in the past 12 months?  (**If YES ask 620 only. If NO ask 621 only**)  YES NO | In the past 12 months would you say that this has happened once, a few times or many times?  One Few Many | Before the past 12 months would you say that this has happened once, a few times or many times?  One Few Many |
| a. | Has your current husband/partner or any other partner ever forced you to have sexual intercourse by threatening you, holding you down or hurting you in some way? | | 1 2 | 1 2 | 1 2 3 | 1 2 3 |
| b. | Have you ever had sexual intercourse when you did not want to because you were afraid that your partner would hurt you or someone you cared about if you refused? | | 1 2 | 1 2 | 1 2 3 | 1 2 3 |
| c. | Have you ever had sexual intercourse when you did not want to because you were afraid that your partner would leave you or take another girlfriend if you refused? | | 1 2 | 1 2 | 1 2 3 | 1 2 3 |
|  | SKIP IF 621 ALL NO  Was the partner who did these things your current partner or another partner? | | | Current 1  Previous 2  Both 3 | | |

**PLEASE LOOK AT QUESTION 210 IF RESPONSE IS NO PARTNER IN PAST 12 MONTHS, GO TO SECTION 7.**

|  | QUESTIONS | CODING CATEGORIES | | | SKIP TO |
| --- | --- | --- | --- | --- | --- |
|  | Thinking back over the past 12 months, would you say that you have been very afraid of your partner never, a few times, many times or most of the time? | | Never  a few times  Many times  Most /all of the time  Refuse to answer | 1  2  3  4  99 |  |
|  | In the past 12 months, how often have children living in your household seen or heard you being beaten by your partner?  Would you say, never, happened few times ,many times or most /all of the time…. | | Never  Few times  Many times  Most /all of the time  Refuse to answer  No children | 1  2  3  4  99  96 | 632 |
|  | In the past 12 months, how often has someone in your household spanked, slapped, kicked, punched or beaten your children up?  Would you say, never, happened few times ,many times or most /all of the time…. | | Never  Few times  Many times  Most /all of the time  Refuse to answer | 1  2  3  4  99 |  |
|  | In the past 12 months, how often has someone in your household hit your children so hard that they had marks or injuries?  Would you say, never, happened few times ,many times or most /all of the time…. | | Never  Few times  Many times  Most /all of the time  Refuse to answer | 1  2  3  4  99 | 632 |
|  | In the past 12 months how often was your husband/partner Hit your children so hard that left marks or were injured?  Would you say, never, happened few times ,many times or most /all of the time…. | | Never  Few times  Many times  Most /all of the time  Refuse to answer | 1  2  3  4  99 |  |
|  | In the past 12 months how often was any person in your household hit your children so hard that left marks or were injured?  Would you say, never, happened few times ,many times or most /all of the time…. | | Never  Few times  Many times  Most /all of the time  Refuse to answer | 1  2  3  4  99 |  |

**If YES to any sub-question in 611, 616 or 621, continue. If ALL NO, SKIP to SECTION 7.**

From your answers I can see that you have had some difficulties with yourcurrent partner or past partners. Now I would like to ask learn more about what you have done to deal with these difficult situations.

|  | **QUESTIONS** | **CODING CATEGORIES** | | | **SKIP TO** |
| --- | --- | --- | --- | --- | --- |
|  | In the past 12 months, during these incidents of violence did you ever fight back physically to defend yourself? | | Yes  No  No partner in the past 12 months | 1  2  3 | 634  636 |
|  | IF YES: READ OUT ANSWERS | | Once  Few times  Many times  Don’t know  Refuse to answer | 1  2  3  96  99 |  |
|  | In the past 12 months did you ever hit or physically mistreated your husband/partner when he was not hitting or physically mistreating you? | | Yes  No | 1  2 | 636 |
|  | IF YES: READ OUT ANSWERS | | Once  Few times  Many times  Don’t know  Refuse to answer | 1  2  3  96  99 |  |
|  | Was there ever a time you were beaten or assaulted by any of your partner(s) while you were pregnant? | | Yes  No  Never been pregnant | 1  2  3 | 639  639 |
|  | In how many pregnancies were you beaten?  USE 96IF NOT KNOWN | | Number [ ] [ ] |  |  |
|  | During the most recent pregnancy where you were beaten, was the perpetrator the father of your child? | | Yes  No | 1  2 |  |

| 639 -640 | | Who have you told about your partner’s behaviour? | | YES NO | FOR EACH MENTIONED, ASK: Have you talked with them within the last 12 months?  YES NO | | |
| --- | --- | --- | --- | --- | --- | --- | --- |
| a. | | Friend | | 1 2 | 1 2 | | |
| b. | | Parents | | 1 2 | 1 2 | | |
| c. | | Brother or sister | | 1 2 | 1 2 | | |
| d. | | Uncle or aunt | | 1 2 | 1 2 | | |
| e. | | Husband/partner’s family | | 1 2 | 1 2 | | |
| f. | | Children | | 1 2 | 1 2 | | |
| g. | | Neighbours | | 1 2 | 1 2 | | |
| h. | | Police | | 1 2 | 1 2 | | |
| i. | | Doctor/health worker | | 1 2 | 1 2 | | |
| j. | | Religious leaders | | 1 2 | 1 2 | | |
| k. | | Counsellor | | 1 2 | 1 2 | | |
| l. | | NGO/women’s organization | | 1 2 | 1 2 | | |
| m. | | Local leader | | 1 2 | 1 2 | | |
| n. | | Member of loan group | | 1 2 | 1 2 | | |
| o. | | BRAC staff | | 1 2 | 1 2 | | |
| p. | | OTHER (specify):_______________________ | | 1 2 | 1 2 | | |
| q. | | No one | | 1 2 |  | | |
| 641 -642 | | Did anyone ever try to help you? | | YES NO | FOR EACH MENTIONED, ASK: Have you talked with them within the last 12 months?  YES NO | | |
| a. | | Friend | | 1 2 | 1 2 | | |
| b. | | Parents | | 1 2 | 1 2 | | |
| c. | | Brother or sister | | 1 2 | 1 2 | | |
| d. | | Uncle or aunt | | 1 2 | 1 2 | | |
| e. | | Husband/partner’s family | | 1 2 | 1 2 | | |
| f. | | Children | | 1 2 | 1 2 | | |
| g. | | Neighbours | | 1 2 | 1 2 | | |
| h. | | Police | | 1 2 | 1 2 | | |
| i. | | Doctor/health worker | | 1 2 | 1 2 | | |
| j. | | ReligiousLeaders | | 1 2 | 1 2 | | |
| k. | | Counsellor | | 1 2 | 1 2 | | |
| l. | | NGO/women’s organization | | 1 2 | 1 2 | | |
| m. | | Local leader | | 1 2 | 1 2 | | |
| n. | | Member of loan group | | 1 2 | 1 2 | | |
| o. | | BRAC staff | | 1 2 | 1 2 | | |
| p. | | OTHER (specify):_______________________ | | 1 2 | 1 2 | | |
| q. | | No one | | 1 2 | 1 2 | | |
| 643-644 | | Did you ever go to any of the following for help?  READ EACH ONE | | YES NO | FOR EACH MENTIONED, ASK: Did you seek help from them within the last 12 months?  YES NO | | |
| a. | | Police | | 1 2 | 1 2 | | |
| b. | | Hospital or health centre | | 1 2 | 1 2 | | |
| c. | | Social services | | 1 2 | 1 2 | | |
| d. | | Legal advice centre | | 1 2 | 1 2 | | |
| e. | | Court | | 1 2 | 1 2 | | |
| f. | | Shelter | | 1 2 | 1 2 | | |
| g. | | Street leader | | 1 2 | 1 2 | | |
| h. | | Women’s organization: Name ,specify_______________ | | 1 2 | 1 2 | | |
| i. | | Priest/Religious leader | | 1 2 | 1 2 | | |
| j. | | Anywhere else? Where? specify__________________ | | 1 2 | 1 2 | | |
|  | Have you ever left, even if only for a night, because of his behaviour? | Yes  No | | | 1  2 | 652 | |
|  | If so, how many times? | Number of times [ ] [ ] | | |  |  | |
|  | Did you leave at least one night in the past 12 months? | Yes  No | | | 1  2 |  | |
|  | How long did you stay away the **last time you left**? | One day  Several days  Months  Left partner | | | 1  2  3  4 |  | |
|  | Where did you go? | Family  Friends/Neighbors  Hotel  Other specify. . . . . . . . . . . . . . . . . . . . . . . . . . . . . | | | 1  2  3  4 |  | |
|  | Did you return? | Yes  No | | | 1  2 | 652 | |
|  | [IF RETURNED] Why did you return? | I did not want to leave Children  Sanctity of Marriage  Family said to return  Did not know how to support myself  Forgave him  He threatened me  Other specify_________________ | | | 1  2  3  4  5  6  7 |  | |
|  | In the past 12 months, overall, how has you being a member of BRAC influenced your ability to cope with your partner’s violence. Would you say that it has: | Not affected  Made it worse  Improved situation  No partner  Refuse to answer  Not in BRAC group | | | 1  2  3  4  96  99 |  | |

| SECTION 7: ABOUT CHILDHOOD |
| --- |
|  |

In this section I would like to ask you a few questions about things you might have seen or things that might have happened to you when you were a child. Some of these questions might be very difficult to answer. I want you to remember that everything you say here is completely confidential and will not be shared with anyone without your permission.

|  | QUESTIONS | CODING CATEGORIES | | | | | | |
| --- | --- | --- | --- | --- | --- | --- | --- | --- |
|  | When you were growing up, during the first 15 years of your life . . . |  | | | | | |  |
| a. | Did your parents/guardians understand your problems and worries? | Yes  No  Don’t know | | | | | | 1  2  96 |
| **b.** | Did you feel you were living in a warm and loving household? | Yes  No  Don’t know | | | | | | 1  2  96 |
| **c.** | Did you live with a household member who was a problem drinker or alcoholic? | Yes  No  Don’t know | | | | | | 1  2  96 |
| d. | Did you live with someone who abused drugs? | Yes  No  Don’t know | | | | | | 1  2  96 |
| **e.** | Did you live with a household member who was sent to jail or prison? | Yes  No  Don’t know | | | | | | 1  2  96 |
| **f.** | Did your father, mother or guardian die? | Yes  No | | | | | | 1  2 |
| g. | Did you see or hear a parent or household member in your home being slapped, kicked, punched or beaten with a fist or objected?  Would you say, never, once, few times, many times? | Never  Once  Few times  Many times | | | | | | 1  2  3  4 |
|  | These next questions are about certain things YOU YOURSELF may have experienced when you were growing up. During the first 15 years of your life, did a parent, or other adult in the household ever…. | Never | Once | Few times | | | Many times | |
| a. | Call you bad words, insult you or put you down?  Would you say never, once , few times, or many times. | 1 | 2 | | 3 | 4 | | |
| b. | Threaten you with physical harm  Would you say never, once or twice, a few times, or many times. | 1 | 2 | | 3 | 4 | | |
| c. | Spank, slap, kick, punch or beat you up?  Would you say never, once , few times, or many times | 1 | 2 | | 3 | 4 | | |
| d. | Hit you so hard that you had marks or were injured  Would you say never, once or twice, a few times, or many times. | 1 | 2 | | 3 | 4 | | |
| e. | Did an adult or person at least 5 years older than you touch or fondle you in a sexual way?  Would you say never, once , few times, or many times | 1 | 2 | | 3 | 4 | | |
| f. | Make you touch their body in a sexual way?  Would you say never, once , few times, or many times | 1 | 2 | | 3 | 4 | | |
| g. | Attempt oral, anal, or vaginal intercourse with you?  Would you say never, once , few times, or many times | 1 | 2 | | 3 | 4 | | |
| h. | Actually have oral, anal, or vaginal intercourse with you?  Would you say never, once , few times, or many times | 1 | 2 | | 3 | 4 | | |
|  | When you were growing up, during the first 15 years of your life, did you see…  READ ANSWERS | Never | Once | | Few times | Many times | | |
| a. | Someone in your community being beaten up? | 1 | 2 | | 3 | 4 | | |
| b. | Somebody get stabbed? | 1 | 2 | | 3 | 4 | | |
| c. | Somebody get shot? | 1 | 2 | | 3 | 4 | | |
| d. | Somebody threatened with a gun or another weapon? | 1 | 2 | | 3 | 4 | | |
| e. | Did a thief or burglar force their way into your house? | 1 | 2 | | 3 | 4 | | |

**PLEASE LOOK AT QUESTION 209. IF RESPONSE IS NO, SKIP TO SECTION 8.**

I would like to ask few questions about things that could have happened to your current/most recent partner /past partner when he was a child.

|  | As far as you know, when your current or most recent partner or past partner was a child, did he live with a household member who was an alcoholic in your household? | Yes  No  Don’t know  No partner | 1  2  96  99 |
| --- | --- | --- | --- |
|  | As far as you know, when your current or most recent partner or past partner was a child, did he live with a household member who was sent to jail | Yes  No  Don’t know  No partner | 1  2  96  99 |
|  | As far as you know, when your current or most recent partner or past partner was a child, did his mother, father or guardian die? | Yes  No  Don’t know  No partner | 1  2  96  99 |
|  | As far as you know, when your current or most recent or past partner was a child, was he beaten himself regularly by someone in his family? | Yes  No  Don’t know  No partner | 1  2  96  99 |
|  | As far as you know, when your current or most recent partner was a child, was he beaten so hard that left marks or injured? | Yes  No  Don’t know  No partner | 1  2  96  99 |

| SECTION 8: ABOUT YOUR COMMUNITY |
| --- |
|  |

I would now like to learn a bit more about the groups and organisations that you are part of in your community.

|  | QUESTIONS | CODING CATEGORIES | | | | | | | | |
| --- | --- | --- | --- | --- | --- | --- | --- | --- | --- | --- |
| 800-801 | Please tell me if you are part of one or several of the following groups. |  | | | If **YES for question 800**, please also tell me if you are simply attending, if you are actively contributing or even leading some of the group activities. | | | | |  |
|  |  | Yes | | No | Member/attends | Active | | Leader | |  |
| a. | Religious group | 1 | | 2 | 1 | 2 | | 3 | |  |
| b. | Ethnic groups/Cultural groups | 1 | | 2 | 1 | 2 | | 3 | |  |
| c. | Economic support groups (non-finance) | 1 | | 2 | 1 | 2 | | 3 | |  |
| d. | *Mtaa* based groups | 1 | | 2 | 1 | 2 | | 3 | |  |
| e. | Celebration/Burial help groups (vikundi vya sherehe/ na kuzikana) | 1 | | 2 | 1 | 2 | | 3 | |  |
| f. | Youth group | 1 | | 2 | 1 | 2 | | 3 | |  |
| g. | Women’s group (Non finance) | 1 | | 2 | 1 | 2 | | 3 | |  |
| h. | School committee | 1 | | 2 | 1 | 2 | | 3 | |  |
| i. | Health committee | 1 | | 2 | 1 | 2 | | 3 | |  |
| j. | Sports group | 1 | | 2 | 1 | 2 | | 3 | |  |
| k. | Credit/finance group | 1 | | 2 | 1 | 2 | | 3 | |  |
| l. | Legal/Professional organization | 1 | | 2 | 1 | 2 | | 3 | |  |
| m. | Advocacy groups | 1 | | 2 | 1 | 2 | | 3 | |  |
| n. | Others (specify) ___________________ | 1 | 2 | | 1 | | 2 | | 3 |  |

|  | QUESTIONS | | CODING CATEGORIES | | |
| --- | --- | --- | --- | --- | --- |
|  | Now I am going to ask you some questions about how the community functions and deals with problems.  Suppose two people in this village/neighborhood had a serious dispute with each other. Who do you think would primarily help resolve the dispute? | No one; people work it out between themselves  Family/household members  Neighbours  Groups’ members  Community leaders  Religious leaders  Judicial leaders  Other (specify) . . . . . . . . . . . . . . . . . . | | 1  2  3  4  5  6  7  8 |  |
|  | In the past 2 years, have you participated in a meeting, march, rally or gathering aiming to raise awareness and mobilize people around an issue that is important in your community? For example, HIV, rights for albinos or women, etc. | Yes  No | | 1  2 |  |
|  | Have you ever been involved in the organization of such a meeting or gathering? | Yes  No | | 1  2 |  |
|  | If **YES,** what kind? | Specify:__________________ | | |  |
|  | People often feel shy about speaking in public. If you were at a community meeting (eg, school committee), how confident are you that you could raise your opinion in public? Would you say….(READ RESPONSES) | Very confident  Confident but would need to be encouraged  Not confident at all  Don’t know | | 1  2  3  96 |  |
|  | Have you spoken out in public in the past 12 months? | Yes  No | | 1  2 |  |
|  | Neighbours often have similar problems (eg, around raising children). How confident do you feel about offering advice to your neighbour or friend? Would you say: (READ RESPONSES) | Very confident  Confident but would need to be encouraged  Not confident at all  Don’t know | | 1  2  3  96 |  |
|  | Have you offered such advice in the last 12 months? | Yes  No | | 1  2 |  |
|  | If you were abused by your husband or partner, how comfortable would you feel seeking support from a trusted friend or neighbor? Would you say: | Very comfortable  Comfortable but would need encouragement  Not comfortable at all  Don’t know | | 1  2  3  99 |  |
|  | How confident are you about your ability to speak up in defense of a woman who has been sexually harassed or violated? Would you say: | Very confident  Confident but would need to be encouraged  Not confident at all  Don’t know | | 1  2  3  99 |  |
|  | How confident are you in your ability to intervene in cases of domestic violence? | Very confident  Confident but would need to be encouraged  Not confident at all  Don’t know | | 1  2  3  99 |  |
|  | Have you intervened in the past 12 months? | Yes  No | | 1  2 |  |

| SECTION 9: ONLY AT FOLLOW UP |
| --- |
|  |

|  | QUESTIONS | CODING CATEGORIES | | SKIP TO |
| --- | --- | --- | --- | --- |
|  | **AT FOLLOW UP ONLY FOR WOMEN IN GROUPS THAT DID NOT RECEIVE GENDER TRAINING** |  |  |  |
|  |  |  |  |  |
|  | Do you know other women who have participated in this study? | Yes  No | 1  2 | 901 |
| a. | Did any of these women participate in gender training sessions? | Yes  No | 1  2 |  |
| b. | Have you discussed issues such as violence against women or the role of women in society with these women? | Yes  No | 1  2 |  |
|  | Have you heard or seen any activities in the community on violence between couples? | Yes  No | 1  2 |  |
| a. | Have you participated in any such activity? | Yes  No | 1  2 |  |
| b. | Do you know who organized these activities? | Name of organization:______________ |  |  |

|  | QUESTIONS | CODING CATEGORIES | | SKIP TO |
| --- | --- | --- | --- | --- |
|  | **AT FOLLOW UP ONLY AND FOR WOMEN WHO ARE IN THE MICROFINANCE PART OF THE TRIAL (COMPONENT A)** |  |  |  |
|  | In the past 2 years, or ever since you joined this study, how many loans did you take from BRAC? | Number [ ] [ ] |  |  |
|  | What was the average size of your loan? | TSH [ ] [ ] [ ] [ ] [ ] [ ] |  |  |
|  | Thinking back to the time before you joined the study, are you taking bigger loans now? | Yes  No | 1  2 |  |
|  | Over the last 2 years, have you continuously been taking loans from BRAC or were there times when you stopped? | Continuously  Stopped at times | 1  2 | 907 |
|  | Why did you stop? | Did not need it any more  Could not pay back the loan  Husband refused to take new loan  Other (specify)……………… | 1  2  3  4 |  |
|  | In the past 2 years, did you take loans from other microfinance providers in addition to the loans from BARC? | Yes  No | 1  2 |  |
|  | If yes, what was the reason? | To pay back the BRAC loan  Needed more money to pay bills  Needed more money to invest in business  Other………………… | 1  2  3  4 |  |

**WHEN YOU ARE DONE**

| **Interview details – END**  Date of interview:  Time interview ended:  Name of interviewer:  Are you the same interviewer as at the beginning? No **Yes**  Comments: |
| --- |

| **Interview closure**  ***Finish one – Respondent has disclosed problems/violence***  I would like to thank you very much for helping us. I appreciate the time that you have taken. I realize that these questions may have been difficult for you to answer, but it is only by hearing from women themselves that we can really understand about their situation, and how to help them.  From what you have told us, I can tell that you have had some very difficult times in your life. No one has the right to threat someone else in that way. However, from what you have told me I can see that you are strong, and have survived through some difficult circumstances.  Here is a list of organizations that provide support, legal advice and counseling services to women in Mwanza. Please do contact them if you would like to talk over your situations with anyone. Their services are free, and they will keep anything that you say private. You can go whenever you feel ready to, either soon or later on. We would be happy to help you contact them, if this would be helpful to you.  ***Finish two – Respondent has not disclosed problems/violence***  I would like to thank you very much for helping us. I appreciate the time that you have taken. I realize that these questions my have been difficult to answer, but it is only by hearing from women themselves that we can really understand about women’s health and experiences in life.  In case you ever hear of another woman who needs help, here is a list of organizations that provide support, legal advice and counseling services to women in Mwanza. Please do contact them if you or any of your friends or relatives need help. Their services are free, and they will keep anything that anyone says to them private. |
| --- |
