# Supplementary material for: Women’s income and risk of intimate partner violence: secondary findings from the MAISHA cluster randomised trial in North-Western Tanzania
Source: BMC Public Health. 2019 Aug 14;19:1108. doi: 10.1186/s12889-019-7454-1 (PMC6694529; doi:10.1186/s12889-019-7454-1)
Supplement: Supplementary file 2 — MAISHA follow-up questionnaire. MAISHA follow-up survey interview (administered 29 months post baseline). (PDF 529 kb) [file 12889_2019_7454_MOESM2_ESM.pdf]

FEMALE PARTICIPANT QUESTIONNAIRE

FOLLOW-UP

CONFIDENTIAL UPON COMPLETION

SECTION 1: ABOUT YOUR HOUSHOLD 4

SECTION 2: ABOUT YOU AND YOUR PARTNER 5

SECTION 3: ABOUT YOU AND YOUR INCOME 8

SECTION 4: ABOUT YOU AND YOUR HEALTH 12

SECTION 5: ABOUT ATTITUDES AND SOCIAL NORMS 16

SECTION 6: ABOUT YOUR RELATIONSHIP 18

SECTION 7: ABOUT CHILDHOOD 24

SECTION 8: ABOUT YOUR COMMUNITY 25

SECTION 9: ONLY AT FOLLOW UP 27

Introduction

Hello, my name is ____________________ , I am from the National Institute for Medical Research/Mwanza Interventions Trial Unit. As you know, you have agreed to take part in the study we are currently conducting in Mwanza. Two years ago we asked you a number of questions about yourself, your household, your relationship and your community. We would now like to ask you some similar questions again. Some of the questions are sensitive, but everything that you say will be kept private, and will not be shared with others. I would like to encourage you to be honest, as there are no right or wrong answers. At any point you can stop the interview, or choose to not answer a question. This will not affect your further involvement in the study. If you find anything that we discuss upsetting, and would like to talk to someone afterwards, we can help with this.

The interview will take two hours or more to complete. For this it is best if we are not interrupted. Is this a good place to talk, or should we go somewhere else where we can talk privately?

Do you have any questions?

BEFORE YOU START

**Identification**	
Branch name	
1. Branch code	**[ ][ ][ ][ ]**
2. Group code	**[ ][ ][ ][ ]**
3. Participant no	**[ ][ ][ ]start with 0**
**4. P**articipant's ID	**A[ ][ ][ ][ ]-[ ][ ][ ][ ]-[ ][ ][ ]**

! PLEASE FILL IN COMPLETE PARTICIPANT ID AT THE BOTTOM OF EVERY PAGE OF THIS QUESTIONNAIRE !

Interview details – START

Date of interview: **[ ][ ]/[ ][ ][ ]/[ ][ ][ ][ ] dd/MMM/yyyy**

Time interview started: **[ ][ ]:[ ][ ]**

Name of interviewer: **[ ][ ][ ]**

SECTION 1: ABOUT YOUR HOUSHOLD

I would like to start by asking you a few questions about your household. When talking of your household, I mean the place and the people that you normally share food with, and sleep with under the same roof.

MAKE SURE THAT ALL QUESTIONS APPLY TO HER HOUSEHOLD AND NOT THE PLACE YOU ARE CURRENTLY CONDUCTING THE INTERVEIW.

	QUESTIONS	CODING CATEGORIES	
100.	Is the house you live in rented, owned by you (either on your own, or with someone else), or owned by someone else in your family?	Rent<br>Own themselves<br>Owned by someone else in family<br>Owned by someone else other than family member<br>Owned together with someone	1<br>2<br>3<br>4<br>5
101.	How many rooms does your household use for sleeping?	[ ][ ]	
102.	What type of toilet is mainly used in your household?	Bush<br>Uncovered pit latrine<br>Covered pit latrine shared<br>Covered pit latrine private<br>VIP latrine shared<br>VIP latrine private<br>Flush toilet shared<br>Flush toilet private<br>Other (specify)…………………………	1<br>2<br>3<br>4<br>5<br>6<br>7<br>8<br>9
103.	What is the main source of lighting?	Electricity<br>Paraffin, kerosene or gas lantern<br>Firewood<br>Candle<br>Other (Specify)…………………………………	1<br>2<br>3<br>4<br>5

SECTION 2: ABOUT YOU AND YOUR PARTNER

I would now like to ask you a few questions about yourself and your partner.

The next few questions are about your current or most recent partner. Please remember that everything you mention will be kept confidential and not share with anyone from your community.

	QUESTIONS	CODING CATEGORIES		SKIP TO
200.	Are you married or currently living with a man, as if married?	YES	1	202
		NO	2	
201.	Have you been in a relationship with a man during the past 12 months?	Yes	1	
		No	2	215
202.	Is this the same man as when we you interviewed two years ago?	YES	1	215
		NO	2	
	If the partner is a different man, ask questions 203 to 214<br><br>The next questions relate to the man that you consider to be your husband or main partner.			
203.	How long have (had) you been in this relationship?<br>GET TO ESTIMATE IF DON'T KNOW EXACTLY.CODE 00 IF MONTH UNKNOWN	Years: [ ][ ] Months: [ ][ ]		
204.	Is (was) your partner married to another woman?	Yes	1	
		No	2	206
		DON'T KNOW	3	206
205.	Are (were) you the first, second, ... wife/partner?<br>CODE 96 IF UNKNOWN	Rank: [ ][ ]		
206.	How old is (was) your partner?<br>CODE 96 IF UNKNOWN	Years: [ ][ ]		
207.	What is(was) your partner's religion?<br><br>CIRCLE ONE	Muslim	1	
		Seventh day Adventist	2	
		Tanzanian Assemblies of God	3	
		Catholic	4	
		Lutheran	5	
		Moravian	6	
		Pentecostal	7	
		African Inland Church	8	
		Don't know	9	
		Other (Specify)::	10	
208.	What is (was) your partner's ethnic background?<br><br>CIRCLE ONE	Msukuma	1	
		Mjita	2	
		Mzinza	3	
		Myiramba	4	
		Mkara/Mikerewe	5	
		Mhaya	6	
		Mjaluo	7	
		Mkuria/Mshashi	8	
		Mchaga	9	
		Mhindi	10	
		Mwarabu	11	
		Don't know	12	
		Other	13	

209.	What is the highest level of education that he completed?<br><br>CIRCLE ONE	Never went to school<br>Primary incomplete<br>Primary complete<br>Secondary incomplete<br>Secondary (Form I-IV)<br>Secondary (Form V-VI)<br>College training after primary/secondary school and before university<br>University<br>Don't know	1<br>2<br>3<br>4<br>5<br>6<br>7<br><br>8<br>9	
210.	Has your partner worked for money during the past 12 months (either employed or self-employed)?	Yes<br>No	1<br>2	 215
211.	Is this work self-employed or is he employed?	Self employed<br>Employed	1<br>2	
212.	Does your partner usually work throughout the year, or does he work seasonally, or only once in a while?	Throughout the year<br>Seasonally/Part Of The Year<br>Once in a while	1<br>2<br>3	
213.	Does your partner have to travel away from home for his work? How long would you say he has been away in the past year (12 months)?:<br>READ RESPONSE CODES CIRCLE ONE	More than half of the year<br>About half of the year<br>Less than half of the year<br>Not all	1<br>2<br>3<br>4	
214.	Over a typical month/week in the past year, how much money does your partner earn?<br>USE 969696 IF DON'T KNOW	Month TSH (estimate):[ ][ ] [ ][ ] [ ][ ]<br>Week TSH (estimate): [ ][ ] [ ][ ] [ ][ ]		

I would like to learn a bit more about the children that you are responsible for, including both your own children and other children that live with you, as well as children living elsewhere. Please remember this information is confidential and when we are using initials of your children it is only to make sure which child we are talking about. This cannot be used to identify your children!
USE THIS SECTION TO BUILD A RAPPORT WITH THE INTERVIEWEE.

CHILDREN THAT RESPONSIBLE FOR<br>Are there children less than 18 months who you are responsible?		Yes<br>No	1<br>2 (If No go to Question 300)
**215-216**	How many children up to the age of 18 are you responsible for including those living in the household and those living elsewhere?	215.<br>No. living with you<br><br>[ ][ ]	216.<br>No. living elsewhere<br><br>[ ][ ]

217-222	I will ask you a little about each child in turn , starting with the eldest child.	START WITH OLDEST.<br>No of child<br>(1-6)	217.	218.	219.	220.	221.	222.
a.	What is their initials or nickname?	FILL IN **ALL** INITIALS						
b.	IS this a boy or a girl?	Female<br>Male	1<br>2	1<br>2	1<br>2	1<br>2	1<br>2	1<br>2
c.	What is their date of birth?	Day<br>Month (MMM)<br>Year<br>(USE 96 FOR ALL IF NOT KNOWN)	[ ][ ]<br>_ _ _<br>[] [] [] [ ]	[ ][ ]<br>_ _ _<br>[] [] [] [ ]	[ ][ ]<br>_ _ _<br>[] [] [] [ ]	[ ][ ]<br>_ _ _<br>[] [] [] [ ]	[ ][ ]<br>_ _ _<br>[] [] []<br>[]	[ ][ ]<br>_ _ _<br>[] [] [] [ ]
d.	How old is he/she?	IF DATE UNKNOWN USE 96.<br>USE 01 IF <1 YEAR	[ ][ ]	[ ][ ]	[ ][ ]	[ ][ ]	[ ][ ]	[ ][ ]
e.	Is [NAME] living with you	No in another household<br>No away at school<br>Yes	1<br><br>2<br>3	1<br><br>2<br>3	1<br><br>2<br>3	1<br><br>2<br>3	1<br><br>2<br>3	1<br><br>2<br>3
f.	Are you the mother?	Yes<br>No - Other woman	1<br>2	1<br>2	1<br>2	1<br>2	1<br>2	1<br>2
g.	Is the father your current partner, or another man?	Current partner<br>Previous partner<br>Other man	1<br>2<br>3	1<br>2<br>3	1<br>2<br>3	1<br>2<br>3	1<br>2<br>3	1<br>2<br>3

h.	Is [NAME] at school?	Never SKIP to J<br>In the past<br>Currently	1<br>2<br>3	1<br>2<br>3	1<br>2<br>3	1<br>2<br>3	1<br>2<br>3	1<br>2<br>3
i.	What is the highest grade they reached	Primary<br>Secondary<br>Nursery<br>college	1<br>2<br>3<br>4	1<br>2<br>3<br>4	1<br>2<br>3<br>4	1<br>2<br>3<br>4	1<br>2<br>3<br>4	1<br>2<br>3<br>4
j.	IF NOT ENROLLED IN SCHOOL: What is the main reason why they aren't in school [NB do not read out answers]	Truancy<br>Pregnancy<br>Marriage<br>School fees<br>Lack of money<br>Illness of child<br>Caring for family members<br>Failed exams<br>Has "finished"<br>Farming/ household chores<br>Too young<br>Other	1<br>2<br>3<br>4<br>5<br>6<br>7<br>8<br>9<br>10<br>11<br>12	1<br>2<br>3<br>4<br>5<br>6<br>7<br>8<br>9<br>10<br>11<br>12	1<br>2<br>3<br>4<br>5<br>6<br>7<br>8<br>9<br>10<br>11<br>12	1<br>2<br>3<br>4<br>5<br>6<br>7<br>8<br>9<br>10<br>11<br>12	1<br>2<br>3<br>4<br>5<br>6<br>7<br>8<br>9<br>10<br>11<br>12	1<br>2<br>3<br>4<br>5<br>6<br>7<br>8<br>9<br>10<br>11<br>12
k.	IF HAVE LEFT SCHOOL:, What age did they leave at?		[ ][ ]	[ ][ ]	[ ][ ]	[ ][ ]	[ ][ ]	[ ][ ]
l.	IF STILL IN SCHOOL: are they currently repeating year?	Yes<br>No	1<br>2	1<br>2	1<br>2	1<br>2	1<br>2	1<br>2

SECTION 3: ABOUT YOU AND YOUR INCOME

I would now like to find out a bit more about the ways that you earn money.

300.	What is the main sources of income for you and your family? CAN ALLOW MULTIPLE RESPONSES	Yes	No	
a	Money from own work	1	2	
b.	Support from husband/partner	1	2	
c.	Support from other relatives	1	2	
d.	Pension	1	2	
e.	Social services/welfare	1	2	
f.	Other (specify)	………………………..		
301.	Have you personally earned money during the past 12 months?	Yes 1<br>No 2		 320
302.	Are you self-employed or do you work for someone else/organisation?	Self employed 1<br>Worked for someone else or an organization 2<br>Both 3		310
303.	(IF WORKING FOR SOMEONE ELSE OR AN ORGANISATION), Who is your main employer?	Relative 1<br>Neighbor 2<br>Friend / acquaintance 3<br>Government 4<br>NGO 5<br>Private company / Someone's business 6<br>Other (Specify):……. 7		
The following questions refer only to work you did while employed by someone else or an organization<br>Skip if not employed by someone else				
304.	Is this a regular or an occasional activity?	Regular 1<br>Occasional 2		
305.	Do you usually work throughout the year, or do you work seasonally, or only once in a while?	Throughout the year 1<br>Seasonally/Part Of The Year 2<br>Once in a while 3		
306.	For how many months in total did you do this activity during the past year? Would you say	1 month or less 1<br>1-6 months 2<br>6-9 months 3<br>9-12 months 4		
307.	Are you paid in cash, given food or other items, or are you not paid at all?	Cash only 1<br>Cash and items 2<br>Items only 3<br>Other 4<br>Not paid 5		 <br>309<br>309<br>309
308.	On a typical working day/week/month how much do you earn from this activity? FILL OUT ONLY ONE, EITHER DAY, WEEK OR MONTH. ENTER 969696 IF DON'T KNOW	Day: [ ] [ ] [] [ ] [] [ ]<br>Week: [ ] [ ] [] [ ] [] [ ]<br>Month: [ ] [ ] [] [ ] [] [ ]		
309.	Do you plan to continue this activity over the next 12 months?	Yes 1<br>No 2<br>Don't know 96		
	If self-employed…			

310.	What kind of business do you run?<br>(MULTIPLE RESPONSES OK)		Yes	No
		a. Selling vegetables	1	2
		b. Selling homemade food	1	2
		c. Running small restaurant	1	2
		d. Sewing or fixing cloths	1	2
		e. Selling cloths	1	2
		f. Brewing	1	2
		g. Running a salon	1	2
		h. Running a shop	1	2
		I. Selling fish	1	2
		j. Other (Specify):……………………….		

311.	Have you been operating this business for more than or less than 12 months?	Less than 12 months 1<br>More than 12 months 2	
312.	Are you mainly responsible for this activity, or are others responsible?	I am mainly responsible 1<br>Others are responsible 2	
313.	How many other household members contributed time, money or ideas to this activity in the past 12 months?<br>ENTER 96 IF DON'T KNOW	Number:[ ] [ ]	
314.	For how many days did you do this activity during the past month?<br>ENTER 96 IF DON'T KNOW	Number: [ ] [ ]	
315.	On a typical working day, how many hours do you work?<br>ENTER 96 IF DON'T KNOW	Hours: [ ] [ ]	
316.	On a typical working day/week/month how much do you earn from this activity?<br>FILL OUT EITHER DAY, WEEK OR MONTH	Day: [ ] [ ] [] [ ] [] [ ]<br>Week: [ ] [ ] [] [ ] [] [ ]<br>Month: [ ] [ ] [] [ ] [] [ ]	
317.	During the past month, how many times did you not earn enough to cover the costs of running your business?	Never 1<br>Once 2<br>Few times 3<br>Many times 4	
318.	Do you employ other people and pay them a wage?	Yes 1<br>No 2	
319.	Of the money you earned over the past month, what proportion did you reinvest to maintain or expand your business?	None of it 1<br>Some of if 2<br>Half of it 3<br>Most of it 4<br>All of it 5	

	QUESTIONS	CODING CATEGO RIES	SKIP TO
	I would now like to ask you some questions about whether you have been using a microfinance provider, such as SACCOS, BRAC, FINCA or others?		
320.	In the past 12 months, Have you ever borrowed money from a microfinance provider, different from BRAC like SACCOS, BRAC, FINCA or others?	Yes 1<br>No 2	 324
321.	Who have you borrowed money from any organization different from BRAc in the past 12 months?	Yes No<br>a. FINCA 1 2<br>b SACCOS 1 2<br>c. PRIDE 1 2<br>d. JIJENGE 1 2<br>e. Other (specify)…………….. 1 2	
322.	Are you still borrowing money from one of these organization?	Yes 1<br>No 2	
323.	About your current loan which was not from BRAC,, how much money did you borrow?<br>USE 0 IN ALL SPACES TO WRITE IF ONLY BRAC LOAN AND NOT OTHER	Amount [ ] [ ][ ] [ ][ ] [ ][ ]	
324.	Are you currently borrowing money from BRAC?	Yes 1<br>No 2	 332
325.	About your current loan you got from BRAC, how much money did you borrow?<br>WRITE 96 IF DON'T KNOW	Amount [ ] [ ][ ] [ ][ ] [ ][ ]	

326.	In the past 12 months, have you been continuously borrowing money from BRAC, or where there times when you did not borrow money from them?	Continuously<br>Interrupted<br>New loan	1<br>2<br>3	328<br><br>328
327.	Why did you interrupt borrowing money from BRAC?	Did not need it any more<br>Could not pay back the loan<br>Husband refused to take new loan<br>Other (specify)……………………………….	1<br>2<br>3<br>4	
328.	How do/did you **primarily** use the money you borrow?<br><br>READ OUT RESPONSES	To build a small business<br>To maintain a business<br>To pay for medical expenses<br>To pay for school expenses<br>To buy food or clothes<br>To pay back another microfinance loan<br>To pay for rent<br>To help other family members<br>Others (specify) ………………………..	1<br>2<br>3<br>4<br>5<br>6<br>7<br>8<br>9	
329.	How confident do you feel about being able to pay this back? Would you say that you feel:<br>Very confident<br>Somewhat confident<br>Not very confident	Very confident<br>Somewhat confident<br>Not very confident	1<br>2<br>3	
330.	How important is the money that you get from BRAC and other microfinance groups? Would you say that it is extremely important, somewhat important, or not very important at all.	Extremely important<br>Somewhat important<br>Not very important	1<br>2<br>3	
331.	In the past 12 months, overall, how has you being a member of BRAC or other microfinance groups influenced your relationship with your partner? Would you say that its has:= had not effect on the relationship, made your relationship more difficult, or improved your relationship?	Not effected the relationship<br>Made your relationship more difficult<br>Improved your relationship<br>Not in a relationship	1<br>2<br>3<br>9	

	QUESTIONS	CODING CATEGORIES			
332.	Nowadays, many families have a hard time making ends meets. I would like to learn more about how your household is coping. During the last 12 months, how many times…<br><br>EMPHASIZE THAT YOU ARE TALKING ABOUT THE PAST **12 MONTHS**<br><br>**In the past 12 months…….**		If **not NEVER**…		
		Never	Once	Few times	Many times
a.	… were you very worried/stressed about your general financial situation.<br>Would you say ,this has happened or never happened?	1	2	3	4
b.	… have you had trouble buying food or other necessities for your family?<br>Would you say ,this has happened or never happened?	1	2	3	4
c.	… have you had to borrow money to pay rent or other bills ?<br>Would you say ,this has happened or never happened?	1	2	3	4

d.	... did any of your family members need to see a doctor but could not because you did not have enough money?<br>Would you say ,this has happened or never happened?	1	2	3	4
e.	... did your children miss days of school because you did not have money for school fees, uniforms or supplies?<br>Would you say ,this has happened or never happened?	1	2	3	4
f.	... have you or any of your own children gone a whole day without eating anything because there was not enough food?<br>Would you say ,this has happened or never happened?	1	2	3	4

	QUESTIONS	CODING CATEGORIES	
333.	During the past 12 months, how important is the money that you yourself bring into the family? Would you say that it is extremely important, somewhat important, or not very important at all.	Extremely important<br>Very important<br>Somewhat important<br>Not very important<br>Don't bring in any money	1<br>2<br>3<br>4<br>5
334.	Would you say that the money that you bring into the household is more than what your husband/partner contributes, less than what he contributes, or about the same as he contributes?	Less than partner<br>Same as Partner<br>More than partner<br>Has no partner	1<br>2<br>3<br>4
335.	Do you think that you would be able to look after yourself and your family on your income alone? Would you say Definitely yes, yes with difficulty, probably not, or definitely not.	Yes definitely<br>Yes with difficulty<br>Probably not<br>Definitely not	1<br>2<br>3<br>4

SECTION 4: ABOUT YOU AND YOUR HEALTH

400	I would like to learn about your approach to life, in general. I am going to read you some statements, and I would like you to say whether they are true or false:<br>If true,<br>Ask somewhat true, true or definitely true<br>If not true,<br>Ask, somewhat false, false, or definitely false	Definitely true	Mostly true	Slightly true	Slightly false	Mostly false	Definitely f false
a.	You cannot think of many ways to get out of a difficult situation when life evolves unexpectedly.	1	2	3	4	5	6
b.	You energetically pursue your goals.	1	2	3	4	5	6
c.	You feel tired most of the time.	1	2	3	4	5	6
d.	There are lots of ways around any problem.	1	2	3	4	5	6
e.	You are easily downed in an argument.	1	2	3	4	5	6
f.	You cannot think of many ways to get the things in life that are important to you.	1	2	3	4	5	6
g.	You worry about your health.	1	2	3	4	5	6
h.	Even when others get discouraged, you know you can find a way to solve the problem.	1	2	3	4	5	6
i.	Your past experiences have not prepared you well for your future.	1	2	3	4	5	6
j.	You've been pretty successful in life.	1	2	3	4	5	6
k.	You usually find yourself worrying about something.	1	2	3	4	5	6
l.	You do not meet the goals that you set for yourself.	1	2	3	4	5	6

I would now like to ask you some questions about your health.

401	During the last 4 weeks, have you been bothered by any of the following problems?	Yes	No
a.	Do you often have headaches?	1	2
b.	Is your appetite poor?	1	2
c.	Do you sleep badly? Like difficulties falling asleep, waking up in the middle of the night more than 3 times or waking up early in the morning and not getting back to sleep.	1	2
d.	Are you easily frightened?	1	2
e.	Do your hands shake?	1	2
f.	Do you feel nervous, tense or worried?	1	2
g.	Is your digestion poor? Like you are often constipated, feel nauseous, or you don't have an appetite.	1	2
h.	Do you have trouble thinking clearly?	1	2
i.	Do you feel unhappy?	1	2
j.	Do you cry more than usual? Like every day or more than once per day, because of problems?	1	2
k.	Do you find it difficult to enjoy your daily activities?	1	2
l.	Do you find it difficult to make decisions?	1	2
m.	Is your daily work suffering?	1	2
n.	Are you unable to play a useful part in life?	1	2
o.	Have you lost interest in things?	1	2
p.	Do you feel that you are a worthless person?	1	2
q.	Has the thought of ending your life been on your mind?	1	2
r.	Do you have uncomfortable feelings in your stomach?	1	2
s.	Are you easily tired?	1	2

402	On the following question we would like to know how satisfied you feel about your life in general.		
a.	Overall, how satisfied are you with life as a whole these days?	Not at all satisfied<br>A little satisfied<br>Somewhat satisfied<br>Completely satisfied	1<br>2<br>3<br>4
	The following question asks in general how you feel the things you do in your life are worthwhile.		
b.	Overall, to what extent do you feel the things you do in your life are worthwhile?	Not at all worthwhile<br>A little valuable<br>Somewhat worthwhile<br>Completely worthwhile	1<br>2<br>3<br>4
	The following questions ask about how you felt yesterday in general.		
c.	Did you feel happy yesterday?	Not at all happy<br>A little bit happy<br>Somewhat happy<br>Completely happy	1<br>2<br>3<br>4
d.	Did you feel worried yesterday?	Not at all worried<br>A little bit worried<br>Somewhat worried<br>Completely worried	1<br>2<br>3<br>4
e.	Did you feel depressed yesterday?	Not at all depressed<br>A little depressed<br>Somewhat depressed	1<br>2<br>3

	Completely depressed	4

I would now like to ask you some questions about your reproductive health. Some of the questions may be embarrassing to answer. Please remember that this information is confidential and you may choose not to answer them.

403	Have you been pregnant since the last interview?<br>IF YES: How many times?	Yes 1<br>No 2<br>Number [ ][ ]	406
404	How many children have you given birth to since the last interview?	Number [ ][ ]<br>If 00 SKIP TO 406	
405	How many of these children are alive?<br>CODE 0 IF NOT ALIVE	Number [ ][ ]	
406	Are you currently using modern contraceptive methods like hormonal contraceptives, loop or alike?	Yes 1<br>No 2<br>Pregnant 3	
407	How many people in total have you had sexual intercourse with in the past year?<br>CODE 99 IF HAS REFUSED TO ANSWER.<br>IF 00 SKIP TO QN 410	Give total number: [ ][ ]	
408	During the last 12 months, have you ever received money or material goods in exchange for sex?	Yes 1<br>No 2<br>No response 99	
409	In the past 12 months, when you had sex, how often did you use a condom? Would you say:<br>READ RESPONSES	Almost always 1<br>Most of the time 2<br>Some times 3<br>Almost never 4	
410	I don't want to know the result, but in the past year, *have you* had an HIV test?	Yes 1<br>No 2<br>No response 99	

Increasingly women and men in Tanzania are drinking alcoholic beverages. If you don't mind, I would like to ask you about yours and your husband/partner's use of alcohol. Please feel free to be open.

**411**	In the past 12 months, have you ever drunk an alcohol-containing beverage? For example beer, wine, local brew, local spirit (eg. Gongo) or other alcoholic beverage?	Yes 1<br>No 2	415
**412**	How often do you have a drink containing alcohol? Would you say:<br>READ RESPONSES:	1 -6 times per year 1<br>2-4 times a month 2<br>2-3 times a week 3<br>4 or more times a week 4	
**413**	On average, how many drinks containing alcohol do you have on a typical day when you are drinking?	1 or 2 1<br>3 or 4 2<br>5 or 6 3<br>7, 8 or 9 4<br>10 or more 5	

414		No	Once per year	Once per every month	Once per every week	Daily, almost [illegible]
a.	Do you use six or more drinks on one occasion?<br>IF YES , READ OUT ANSWERS	1	2	3	4	5
b.	In the past 12 months, have you found that you were not able to stop drinking once you had started?<br>IF YES , READ OUT ANSWERS	1	2	3	4	5
c.	In the past 12 months ,have you failed to do what was normally expected of you because of drinking?<br>IF YES , READ OUT ANSWERS	1	2	3	4	5

d.	In the past 12 months, have have you needed a first drink in the morning to get yourself going after a heavy drinking session?<br>IF YES , READ OUT ANSWERS	1	2	3	4	5	
e.	In the past 12 months, have you had a feeling of guilt or remorse after drinking?<br>IF YES , READ OUT ANSWERS	1	2	3	4	5	
f.	In the past 12 months, have you been unable to remember what happened the night before because of your drinking?<br>IF YES , READ OUT ANSWERS	1	2	3	4	5	
g.	Have you or someone else been injured because of your drinking – either in the past 12 months, or before this?	Yes, during the past 12 months 1<br>Yes, but not in the last year 2<br>No 3					
h.	Has a relative, friend, doctor, or other health care worker been concerned about your drinking or suggested you cut down?	Yes, during the past 12 months 1<br>Yes, but not in the last year 2<br>No 3					
415	Does your partner drink alcohol?	Yes 1<br>No 2<br>No partner 3					417<br>Go to section 5
416	In the past 12 months, how often have you seen your partner intoxicated (drunk)?	Never 1<br>Once 2<br>Few times 3<br>Many times 4					
417	In the past 12 months, how often have you seen your partner physically fight with other people you are not living with?	Never 1<br>Once 2<br>Few times 3<br>Many times 4					

SECTION 5: ABOUT ATTITUDES AND SOCIAL NORMS

In this community and elsewhere, people have different ideas about families and what is acceptable behavior for men and women in the home. We would like to know your views on what is acceptable and what you think other people in your community believe.

	QUESTIONS	CODING CATEGORIES			
500	I am going to make a number of statements about men and women in general. When I read the following statements can you please indicate how much you personally agree or disagree.<br><br>READ STATEMENTS, THEN ASK IF AGREE OR DISAGREE. THEN ASK IF AGREE OR STRONGLY AGREE OR DISAGREE OR STRONGLY DISAGREE	In your personal opinion…			
		I strongly agree	I agree	I disagree	I strongly disagree
a.	A couple should decide together things that affect the health and well-being of the family	1	2	3	4
b.	It's a wife's obligation to have sex with her husband even if she doesn't want to	1	2	3	4
c.	It **must** be the man who is the primary provider for the family.	1	2	3	4
d.	Women should have the same right as men to study and to work outside of the home	1	2	3	4
e.	A woman should obey her husband's wishes even if she disagrees.	1	2	3	4
f.	Even healthy relationships can include hitting each other as long as the partners love each other.	1	2	3	4
g.	It is perfectly acceptable for women to work outside the home to help support the family economically	1	2	3	4
h.	The leadership of a community should be largely in the hands of men.	1	2	3	4
i.	Children and men would benefit, if fathers were more involved in caring for their children	1	2	3	4
j.	Sons in the family should be given more encouragement to go to school than daughters	1	2	3	4
k.	It is natural and right that men have more power than woman in the family	1	2	3	4
l.	Women could take on many of the roles of men, if men were willing to share power.	1	2	3	4
501	People have different opinions about whether there are situations where a man can be violent towards his partner. When I read the following statements can you please say whether you personally AGREE OR DISAGREE,<br><br>PROBE WHERE NEEDED.<br><br>READ SENTENCE AND ASK IF AGREE , OR STRONGLY AGREE,<br>THEN, ASK IF DISAGREE or STRONGLY DISAGREE	In your personal opinion…			
		I strongly agree	I agree	I disagree	I strongly disagree
a.	A man has a good reason to hit his wife if **she** does not complete her household work to his satisfaction	1	2	3	4
b.	A man has a good reason to hit his wife if **she** disobeys him	1	2	3	4
c.	A man have good reason to hit his wife if **she** refuses to have sexual intercourse with him	1	2	3	4

d.	A man does not have any reason to hit his wife in any way	1	2	3	4
e.	A man has a good reason to hit his wife if **she** protests because he has other girlfriends	1	2	3	4
f.	A man has a good reason to hit his wife if **he** suspects that she is unfaithful in marriage	1	2	3	4
g.	A man has a good reason to hit his wife if he finds out that she has been unfaithful in marriage	1	2	3	4
h.	A woman should tolerate violence in order to keep her family together	1	2	3	4
i.	Violence between husband and wife is a private matter and others should not intervene	1	2	3	4
j.	People should do everything they can to support a woman who has left an abusive husband	1	2	3	4
k.	A woman who is raped should not tell anyone, or she will be blamed for the attack	1	2	3	4
l.	People have a duty to intervene if they hear or see a woman being hit	1	2	3	4
m.	A woman who abandons her abusive husband brings shame upon her family	1	2	3	4

**502**	Now I would like to ask you about your opinion of certain types of behaviours. Please tell me whether or not you consider the following actions a form of violence in all cases, in some cases, or never. Please feel free to speak honestly.<br><br>Would you say that it is violence if:	YES	In some cases	NO
a.	A parent slaps a child for disrespecting his elders	1	2	3
b.	A man hits his wife because she has done something wrong, but leaves no bruises/marks	1	2	3
c.	A wife repeatedly belittles and humiliates her husband in public	1	2	3
d.	A parent yells at a child to stay out of the street	1	2	3
e.	A woman refuses to have sex with her husband for a week	1	2	3
f.	A man hits a woman during an argument but then apologizes	1	2	3
g.	A girl gets a boy sexually aroused but does not allow him to go further	1	2	3
h.	A man refuses to give his wife money to sustain the family even when he has money for other things	1	2	3
i.	A man threatens to hit a woman but does not actually hit her	1	2	3
j.	A man forces a woman he does not know to have sex	1	2	3
k.	A man provides for his family but keeps part of his earnings for himself	1	2	3
l.	A husband forces his wife have sex even though she does not want to	1	2	3

SECTION 6: ABOUT YOUR RELATIONSHIP

IF QUESTION 201 IS NO PARTNER IN PAST 12 MONTHS, START AT 607.

When two people marry, live together or are in a relationship, they usually share both good and bad moments. I would now like to ask you some question about your current and past relationships and how your husband/partner treats (treated) you. If anyone interrupts us I will change the topic of conversation. I would again like to assure you that your answers will be kept secret, and that you do not have to answer any question that you do not want to. May I continue?

	QUESTIONS	CODING CATEGORIES			
600.	During the last 12 months, did you and your partner discuss the following topics together		If happed fill the answer in the shaded area		
		Never	Once	Few times	Many times
a.	... things that happened to you during the day? Would you say never, once, a few times, or many times?	1	2	3	4
b.	... things that happened to him in the day?<br>Would you say never, once, a few times, or many times?	1	2	3	4
c.	... your worries or feelings?<br>Would you say never, once, a few times, or many times?	1	2	3	4
d.	... his worries or feelings?.<br>Would you say never, once, a few times, or many times?	1	2	3	4
601.	During the last 12 month, did your partner ever...		If yes, how often?		
a.	... ask you for your advice to resolve a problem he was facing?<br>Would you say never, once, a few times, or many times?	1	2	3	4
b.	... followed your advice to resolve a problem he was facing?<br>Would you say never, once, a few times, or many times?	1	2	3	4
c.	... helped you in finding work?<br>Would you say never, once, a few times, or many times?	1	2	3	4
d.	... encouraged you to participate in something outside of the home that was only for *your* benefit?<br>Would you say never, once, a few times, or many times?	1	2	3	4
e.	... made you feel appreciated.<br>Would you say never, once, a few times, or many times?	1	2	3	4

602.	How confident do you feel to assert your own opinion if it is different from that of your husband?<br>READ RESPONSES	Very confident<br>Confident but would need to be encouraged<br>Not confident at all<br>Don't know	1<br>2<br><br>3<br>96
603.	Have you asserted your opinion in the past 12 months?	No<br>Yes	2<br>1
604.	How comfortable would you feel in resisting efforts by your husband to control aspects of your life like who you see and how you spend your money?<br>READ ANSWERS	Very comfortable<br>Comfortable but would need encouragement<br>Not comfortable at all<br>Don't know	1<br>2<br><br>3<br>96

605.	No matter how well a couple gets along, there are times when they disagree. **In your relationship with your current / most recent partner....**	Yes	No		
a.	... would you say that you quarreled in the past 12 months?<br>(SKIP TO QUESTION 607 IF ANSWER IS NO)	1	2		
	How often have you quarreled about:<br>IF HAPPENNED, HAS HAPPENED ONCE, FEW TIMES OR MANY TIMES?	Never	Once	Few times	Many times

b.	Accusations that you are not fulfilling your responsibilities as wife and mother	1	2	3	4
c.	His inability or unwillingness to provide for the family	1	2	3	4
d.	Other issues around money and division of resources in the family	1	2	3	4
e.	His drinking/gambling or drug use	1	2	3	4
f.	Your drinking	1	2	3	4
g.	Concerns about outside partners or accusations of infidelity	1	2	3	4
h.	Your refusal to have sex	1	2	3	4
i.	Other issues around sex (frequency, condom use, etc)	1	2	3	4
j.	You disobeying your partner or treating him disrespectfully.	1	2	3	4
k.	Him treating you or your children disrespectfully.	1	2	3	4
l.	Him being unhappy that you had taken a loan from a microfinance organization.	1	2	3	4

606.	In the past 12 months when you have argued with your partner, how generally did you react?<br><br>Would you say that you did the following activities never, once, a few times or many times:	If happed fill the answer in the shaded area			
		Never	Once	Few times	Many times
a.	Expressed how you felt in a calm and respectful way	1	2	3	4
b.	Tried to see your partner's side and listened carefully to what he had to say.	1	2	3	4
c.	You brought in or tried to bring in someone to help settle things.	1	2	3	4
d.	When you felt the argument got too heated, you left so that you and your partner had time to calm down.	1	2	3	4
e.	You started to make complaints about things unrelated to your initial argument.	1	2	3	4
f.	You insulted or swore at him.	1	2	3	4
g.	You yelled	1	2	3	4
h.	You pushed, shaked or pulled him	1	2	3	4

607-608	I am now going to ask you about some situations that are true for many women. Thinking about your (current or most recent or past) husband/partner, would you say it is generally true that he:	607.		608.<br>ONLY ASK IF 'YES' IN 607<br>Has this happened in the past 12 months?	
		Yes	No	Yes	No
a.	Tries to keep you from seeing your friends	1	2	1	2
b.	Tries to restrict contact with your family of birth	1	2	1	2
c.	Insists on knowing where you are at all times	1	2	1	2
d.	Is jealous and gets angry if you speak with another man	1	2	1	2
e.	Is often suspicious that you are unfaithful	1	2	1	2
609 - 610	Thinking about your (current or most recent/past) husband/partner, would you say it is generally true that he:	609.		610.<br>ONLY ASK IF 'YES' IN 609<br>Has this happened in the past 12 months?	
a.	Refuses to give you enough money for household expenses, even when he has money for other things?	1	2	1	2
b.	Takes money that you have earned away from you	1	2	1	2
c.	Makes important financial decisions without consulting you	1	2	1	2

611 – 614	The next questions are about things that happen to many women, and that your current partner, or any other partner may have done to you.<br><br>Has your current husband/partner, or any other partner ever....	611.<br>**(If YES continue with 612**<br>**If NO go to 611 next sub question, if ALL NO skip to 616)**		612.<br>Has this happened in thepast 12 months?<br>**(If YES ask 613 only. If NO ask 614 only)**		613.<br>In the past 12 months would you say that this has happened once, a few times or many times?			614.<br>Before the past 12 months would you say that this has happened once, a few times or many times?		
		YES	NO	YES	NO	One	Few	Many	One	Few	Many
a.	Insulted you or made you feel bad about yourself?	1	2	1	2	1	2	3	1	2	3

b.	Belittled or humiliated you in front of other people?	1 2	1 2	1 2 3	1 2 3
c.	Done things to scare or intimidate you on purpose (e.g. by the way he looked at you, by yelling and smashing things)?	1 2	1 2	1 2 3	1 2 3
d.	Verbally threatened to hurt you or someone you care about?	1 2	1 2	1 2 3	1 2 3
615.	SKIP IF 611 ALL NO<br>Was the person who insulted, humiliated, scared or threatened you your current or a previous partner?	Current 1<br>Previous 2<br>Both 3			

616 - 619	Has <u>your current partner or any other partner</u> ever….	616.<br>**(If YES continue with 617**<br>**If NO go next 616 sub question if ALL NO skip to 621)**<br>YES NO	617.<br>Has this happened <u>in the past 12 months?</u><br>**(If YES ask 618 only. If NO ask 619 only)**<br>YES NO	618.<br><u>In the past 12 months</u> would you say that this has happened once, a few times or many times?<br>One Few Many	619.<br><u>Before the past 12 months</u> would you say that this has happened once, a few times or many times?<br>One Few Many
a.	Slapped you or thrown something at you that could hurt you?	1 2	1 2	1 2 3	1 2 3
b.	Pushed you or shoved you or pulled your hair?	1 2	1 2	1 2 3	1 2 3
c.	Hit you with his fist or with something else that could hurt you?	1 2	1 2	1 2 3	1 2 3
d.	Kicked you, dragged you or beaten you up?	1 2	1 2	1 2 3	1 2 3
e.	Choked or burnt you on purpose?	1 2	1 2	1 2 3	1 2 3
f.	Threatened to use or actually used a gun, knife or other weapon against you?	1 2	1 2	1 2 3	1 2 3
620.	SKIP IF 616 ALL NO<br>Was the person who did these things to you your current partner, or a previous partner?	Current 1<br>Previous /PAST 2<br>Both 3			
621 - 624		621.<br>**(If YES continue with 622.**<br>**If NO go to next 621 sub question, if ALL NO skip to 626)**<br>YES NO	622.<br>Has this happened <u>in the past 12 months</u>?<br>**(If YES ask 623 only. If NO ask 624 only)**<br>YES NO	623.<br><u>In the past 12 months</u> would you say that this has happened once, a few times or many times?<br>One Few Many	624.<br><u>Before the past 12 months</u> would you say that this has happened once, a few times or many times?<br>One Few Many
a.	Has <u>your current husband/partner or any other partner</u> ever forced you to have sexual intercourse by threatening you, holding you down or hurting you in some way?	1 2	1 2	1 2 3	1 2 3
b.	Have you ever had sexual intercourse when you did not want to because you were afraid that your partner would hurt you or someone you cared about if you refused?	1 2	1 2	1 2 3	1 2 3
c.	Have you ever had sexual intercourse when you did not want to because you were afraid that your partner would leave you or take another girlfriend if you refused?	1 2	1 2	1 2 3	1 2 3
625.	SKIP IF 621 ALL NO<br>Was the partner who did these things your current partner or another partner?	Current 1<br>Previous 2<br>Both 3			

626	In the past 12 months, during these incidents of violence did you ever fight back physically to defend yourself?	Yes<br>No<br>No partner in the past 12 months	1<br>2<br>3	 Skip to 628<br>Skip to 632
627	IF YES: READ OUT ANSWERS	Once<br>Few times<br>Many times<br>Don't know<br>Refuse to answer	1<br>2<br>3<br>4<br>5	

628	In the past 12 months did you ever hit or physically mistreated your husband/partner when he was not hitting or physically mistreating you?	Yes<br>No	1<br>2	 Skip to 630
629	IF YES: READ OUT ANSWERS	Once<br>Few times<br>Many times<br>Don't know<br>Refuse to answer	1<br>2<br>3<br>4<br>5	

	QUESTIONS	CODING CATEGORIES		
630	Thinking back over the past 12 months, would you say that you have been very afraid of your partner never, a few times, many times or most of the time?	Never<br>a few times<br>Many times<br>Most /all of the time<br>Refused to answer	1<br>2<br>3<br>4<br>99	
631	In the past 12 months, how often have children living in your household seen or heard you being beaten by your partner?<br>Would you say, never, happened few times, many times or most /all of the time....	Never<br>Few times<br>Many times<br>Most /all of the time<br>Refuse to answer<br>No children	1<br>2<br>3<br>4<br>99<br>96	

If YES FOR ANY OF QUESTIONS 611 or 616 or 621, CONTINUE. IF ALL RESPONSES FOR THESE QUESTIONS ARE NO GO TO QUESTION 646

From your responses I can see that you have had experienced difficult moments with your current or past male partner. Now I would like to ask you about what you did to deal with that difficult experience

632 -633	Who have you told about your partner's behaviour?	632<br>YES	 NO	633<br>FOR EACH MENTIONED, ASK: Have you talked with them within the last 12 months?<br>YES	 NO
a.	Friend	1	2	1	2
b.	Parents	1	2	1	2
c.	Brother or sister	1	2	1	2
d.	Uncle or aunt	1	2	1	2
e.	Husband/partner's family	1	2	1	2
f.	Children	1	2	1	2
g.	Neighbours	1	2	1	2
h.	Police	1	2	1	2
i.	Doctor/health worker	1	2	1	2
j.	Religious leaders	1	2	1	2
k.	Counsellor	1	2	1	2
l.	NGO/women's organization	1	2	1	2
m.	Local leader	1	2	1	2
n.	Member of loan group	1	2	1	2
o.	BRAC staff	1	2	1	2
p.	OTHER (specify):_______________________	1	2	1	2
q.	No one	1	2		
634 -635	Did anyone ever try to help you?	634<br>YES	 NO	635<br>FOR EACH MENTIONED, ASK: Have you talked with them within the last 12 months?<br>YES	 NO
a.	Friend	1	2	1	2
b.	Parents	1	2	1	2
c.	Brother or sister	1	2	1	2
d.	Uncle or aunt	1	2	1	2
e.	Husband/partner's family	1	2	1	2
f.	Children	1	2	1	2
g.	Neighbours	1	2	1	2

h.	Police	1	2	1	2
i.	Doctor/health worker	1	2	1	2
j.	ReligiousLeaders	1	2	1	2
k.	Counsellor	1	2	1	2
l.	NGO/women's organization	1	2	1	2
m.	Local leader	1	2	1	2
n.	Member of loan group	1	2	1	2
o.	BRAC staff	1	2	1	2
p.	OTHER (specify):_______________________	1	2	1	2
q.	No one	1	2	1	2
636-637	Did you ever go to any of the following for help?<br>READ EACH ONE	636<br>YES	 NO	637<br>FOR EACH MENTIONED, ASK: Did you seek help from them within the last 12 months?<br>YES	 NO
a.	Police	1	2	1	2
b.	Hospital or health centre	1	2	1	2
c.	Social services	1	2	1	2
d.	Legal advice centre	1	2	1	2
e.	Court	1	2	1	2
f.	Shelter	1	2	1	2
g.	Street leader	1	2	1	2
h.	Women's organization: Name ,specify_______________	1	2	1	2
i.	Priest/Religious leader	1	2	1	2
j.	Anywhere else? Where? specify__________________	1	2	1	2

**638**	Have you ever left, even if only for a night, because of his behaviour?	Yes<br>No	1<br>2	 645
**639**	If so, how many times?	Number of times [ ] [ ]		
**640**	Did you leave at least one night in the past 12 months?	Yes<br>No	1<br>2	 645
**641**	How long did you stay away the **last time you left**?	One day<br>Several days<br>Months<br>Left partner	1<br>2<br>3<br>4	
**642**	Where did you go?	Family<br>Friends/Neighbors<br>Hotel<br>Other specify.	1<br>2<br>3<br>4	
**643**	Did you return?	Yes<br>No	1<br>2	 645
**644**	[IF RETURNED] Why did you return?	I did not want to leave Children<br>Sanctity of Marriage<br>Family said to return<br>Did not know how to support myself<br>Forgave him<br>He threatened me<br>Other specify_________________	1<br>2<br>3<br>4<br>5<br>6<br>7	
**645**	In the past 12 months, overall, how has your being a member of BRAC influenced your ability to cope with your partner's violence. Would you say that it has:	Not affected<br>Made it worse<br>Improved situation<br>No partner<br>Refuse to answer<br>Not in BRAC group	1<br>2<br>3<br>4<br>96<br>99	

Many women feel uncomfortable discussing these experiences and may not want to disclose everything about their relationships and their experiences. There may be other reasons for a woman not wishing to disclose the information I have just asked you about and that is ok – you do not have to answer questions if you do not want to. I would like to ask you some questions to understand how you feel compared to the last time you were interviewed.<br>(SHOW DATE SHE WAS INTERVIEWED)			

646	Compared to the last time you were interviewed, do you feel more or less comfortable discussing your relationship, in particular the experiences we have just talked about?	More comfortable<br>About the same (the same as when last time she was interviewed)<br>Less comfortable	1<br>2<br>3	
647	Last time you were interviewed, would you say that you reported these experiences accurately?	Yes – reported everything<br>No –did not report everything<br>Not sure/can't remember	1<br>2<br>3	
648	Last time you were interviewed, were there some experiences that you felt you did not wish to discuss or disclose?	Yes – I did not wish to discuss/disclose some experiences<br>No – I did wish to discuss/disclose all experiences<br>Don't remember	1<br>2<br>3	
649	Thinking about the experiences we have just discussed about quarrels/conflicts, would you say that compared to the last time you were interviewed, that these experiences are the same, they are less frequent or more frequent?	Less frequent<br>About the same<br>More frequent	1<br>2<br>3	Skip to 651
650	If the woman indicates a change either less frequent or more frequent – ask her…<br>Do you think you this is because you participated in the MAISHA?	Yes<br>No<br>Do not know	1<br>2<br>3	

651	**If she answered SHE HAS NO CHILD on question 631, go to section 7**<br><br>Adults use certain ways to teach children the right behavior or to address a behavior problem. I will read various methods that are used. Please tell me if you, your partner or another adult in the household has used this method with any of the children in your household in the past month. Indicate one or more of You, Partner, Other	Participant		Your partner or other adult in your household	
		Yes	No	Yes	No
a.	Took away privileges, forbade something they liked or did not allow him/her to leave the house?	1	2	1	2
b.	Explained why your child's behaviour was wrong?	1	2	1	2
c.	Shook him/her	1	2	1	2
d.	Shouted, yelled at or screamed at him/her	1	2	1	2
e.	Gave him/her something else to do	1	2	1	2
f.	Spanked, hit or slapped him/her on the bottom with bare hand	1	2	1	2
g.	Hit him/her on the bottom or elsewhere on the body with something like a belt, hairbrush, or other hard object	1	2	1	2
h.	Called him/her dumb, lazy or another name like that	1	2	1	2
i.	Hit or slapped him/her on the face, head or ears	1	2	1	2
j.	Hit or slapped him/her on the hand, arm or leg	1	2	1	2
k.	Beat him/her up, that is hit him/her over and over as hard as one could	1	2	1	2
l.	Do you believe that in order to bring up, raise, or educate a child properly, the child needs to be physically punished?	1	2	1	2

SECTION 7: ABOUT CHILDHOOD

Check question 201 – if NO,

QUESTIONS	CODING CATEGORIES

PLEASE LOOK AT QUESTION 201. IF RESPONSE IS NO – ASK QUESTIONS 700 TO 704 ABOUT HER CURRENT PARTNER, IF YES, SKIP TO SECTION 8.

I would like to ask few questions about things that could have happened to your current when he was a child.

700.	As far as you know, when your current was a child, did he live with a household member who was an alcoholic in your household?	Yes<br>No<br>Don't know<br>No partner	1<br>2<br>96<br>99
701.	As far as you know, when your current was a child, did he live with a household member who was sent to jail	Yes<br>No<br>Don't know<br>No partner	1<br>2<br>96<br>99
702.	As far as you know, when your current was a child, did his mother, father or guardian die?	Yes<br>No<br>Don't know<br>No partner	1<br>2<br>96<br>99
703.	As far as you know, when your current partner was a child, was he beaten himself regularly by someone in his family?	Yes<br>No<br>Don't know<br>No partner	1<br>2<br>96<br>99
704.	As far as you know, when your current partner was a child, was he beaten so hard that left marks or injured?	Yes<br>No<br>Don't know<br>No partner	1<br>2<br>96<br>99

SECTION 8: ABOUT YOUR COMMUNITY

I would now like to learn a bit more about the groups and organisations that you are part of in your community.

	QUESTIONS	CODING CATEGORIES				
800-801	Please tell me if you are part of one or several of the following groups.	800.		801. If **YES for question 800**, please also tell me if you are simply attending, if you are actively contributing or even leading some of the group activities.		
		Yes	No	Member /attends	Active	Leader
a.	Religious group	1	2	1	2	3
b.	Ethnic groups/Cultural groups	1	2	1	2	3
c.	Economic support groups (non-finance)	1	2	1	2	3
d.	*Mtaa* based groups	1	2	1	2	3
e.	Celebration/Burial help groups (vikundi vya sherehe/ na kuzikana)	1	2	1	2	3
f.	Youth group	1	2	1	2	3
g.	Women's group (Non finance)	1	2	1	2	3
h.	School committee	1	2	1	2	3
i.	Health committee	1	2	1	2	3
j.	Sports group	1	2	1	2	3
k.	Credit/finance group	1	2	1	2	3
l.	Legal/Professional organization	1	2	1	2	3
m.	Advocacy groups	1	2	1	2	3
n.	Others (specify) ___________________	1	2	1	2	3

	QUESTIONS	CODING CATEGORIES	
802.	Now I am going to ask you some questions about how the community functions and deals with problems.<br>Suppose two people in this village/neighborhood had a serious dispute with each other. Who do you think would primarily help resolve the dispute?	No one; people work it out between themselves<br>Family/household members<br>Neighbours<br>Groups' members<br>Community leaders<br>Religious leaders<br>Judicial leaders<br>Other (specify)	1<br>2<br>3<br>4<br>5<br>6<br>7<br>8
803.	In the past 2 years, have you participated in a meeting, march, rally or gathering aiming to raise awareness and mobilize people around an issue that is important in your community? For example, HIV, rights for albinos or women, etc.	Yes<br>No	1<br>2 (Skip to 806)
804.	Have you ever been involved in the organization of such a meeting or gathering?	Yes<br>No	1<br>2 (Skip to 806)
805.	If **YES,** what kind?	Specify:___________________	
806.	People often feel shy about speaking in public. If you were at a community meeting (eg, school committee), how confident are you that you could raise your opinion in public? Would you say....(READ RESPONSES)	Very confident<br>Confident but would need to be encouraged<br>Not confident at all<br>Don't know	1<br>2<br>3<br>99
807.	Have you spoken out in public in the past 12 months?	Yes<br>No	1<br>2
808.	Neighbours often have similar problems (eg, around raising children). How confident do you feel about offering advice to your neighbour or friend? Would you say: (READ RESPONSES)	Very confident<br>Confident but would need to be encouraged<br>Not confident at all<br>Don't know	1<br>2<br>3<br>99

809.	Have you offered such advice in the last 12 months?	Yes<br>No	1<br>2
810.	If you were abused by your husband or partner, how comfortable would you feel seeking support from a trusted friend or neighbor? Would you say:	Very comfortable<br>Comfortable but would need encouragement<br>Not comfortable at all<br>Don't know	1<br>2<br>3<br>99
811.	How confident are you about your ability to speak up in defense of a woman who has been sexually harassed or violated? Would you say:	Very confident<br>Confident but would need to be encouraged<br>Not confident at all<br>Don't know	1<br>2<br>3<br>99
812.	How confident are you in your ability to intervene in cases of domestic violence?	Very confident<br>Confident but would need to be encouraged<br>Not confident at all<br>Don't know	1<br>2<br>3<br>99
813.	Have you intervened in the past 12 months?	Yes<br>No	1<br>2

SECTION 9: ONLY AT FOLLOW UP

Before finishing the interview, I would like to ask few questions about gender training provided as part of this MAISHA project

	QUESTIONS	CODING CATEGORIES	SKIP TO
	**AT FOLLOW UP ONLY FOR WOMEN IN GROUPS THAT DID NOT RECEIVE GENDER TRAINING**		
900.	Have you attended any of the MAISHA gender training sessions?	Yes 1<br>No 2	 903
901	Ten gender training sessions were delivered as part of MAISHA study. How many sessions did you attend? Katika utafiti wa MAISHA mafunzo ya jinsia yalitolewa katika vipindi kumi. Je, wewe ulihudhuria vipindi vingapi?<br><br>Please think carefully so you can give me a correct answer	NUMBER OF SESSIONS[ ] [ ] Write 99 if don't remember	
902.	Which session did you find the most helpful for you personally and for your relationship?<br>(Indicate all sessions she remembers and found helpful)<br><br>Is there anything else that the training helped you with?<br>1 Yes 2 No<br><br>If NO end of probe		
903	Since joining the MAISHA study, have you discussed issues such as violence against women or the role of women in society with other women taking part in MAISHA?	Never 1<br>A few times 2<br>Many times 3<br>All the time 4<br>Refused to answer 5	
904	Since joining the MAISHA study, have you discussed issues such as violence against women or the role of women in society with other women who are NOT taking part in MAISHA?	Never 1<br>A few times 2<br>Many times 3<br>All the times 4<br>Refused to answer 5	
905	Would you like for us to contact you in future about various aspects regarding MAISHA study?	Yes 1<br><br>No 2	

WHEN YOU ARE DONE

Interview details – END

Date of interview:

Time interview ended:

Name of interviewer:

Are you the same interviewer as at the beginning? No **Yes**

Comments:

Survey of men

The aim of the MAISHA study is to explore ways to improve relationships, and health more generally. As you know, we have been working with groups of women receiving microfinance. Now we are interested in learning about men's views about healthy relationships. We are planning to conduct a short interview with the partners of women taking part in MAISHA. During the interview, we will ask the man questions about himself, his health andhis personal experiences in relationships. We would like to know if you agree to your partner being contacted by a male member of the MAISHA team and invited to take part in the study. We will **not** contact him unless you agree. If you agree to us contacting your partner and then change your mind, that is fine – just call us on <NSERT NUMBER>to let us know.

If your partnerconsents to take part in the study, he will be asked to attend one interviewwhich will be conducted in about one month from now at a time and place that is convenient for him. The interview will be conducted by a male interviewer and take about one hour.

If you wish, you can speak to your partner first about the study to find out if he would be interested in taking part and if he agrees to a member of the study team contacting him. Alternatively, if you are happy to give me your partner's mobile number and contact details, one of the male interviewers can contact your partner directly in about one month to arrange a time to meet to discuss the study. We will inform you just before we contact him in case you change your mind.

Do you think your partner might be interested in taking part? 1. Yes 2. No 3. Don't know 4. No answer

If yes,
Would you prefer to talk to your partner first? 1. Yes 2. No 3. Don't know 4. No answer

Do you agree to a member of the MAISHA team contacting him in about one month?
1. Yes 2. No 3. Don't know 4. No answer

If yes, please complete details below:

Partner's name:

Partner's mobile number:

Other partner contact information (e.g. work place):

Interview closure

Finish one – Respondent <u>has disclosed</u> problems/violence
I would like to thank you very much for helping us. I appreciate the time that you have taken. I realize that these questions may have been difficult for you to answer, but it is only by hearing from women themselves that we can really understand about their situation, and how to help them.

From what you have told us, I can tell that you have had some very difficult times in your life. No one has the right to threat someone else in that way. However, from what you have told me I can see that you are strong, and have survived through some difficult circumstances.
Here is a list of organizations that provide support, legal advice and counseling services to women in Mwanza. Please do contact them if you would like to talk over your situations with anyone. Their services are free, and they will keep anything that you say private. You can go whenever you feel ready to, either soon or later on. We would be happy to help you contact them, if this would be helpful to you.

Finish two – Respondent <u>has not disclosed</u> problems/violence
I would like to thank you very much for helping us. I appreciate the time that you have taken. I realize that these questions my have been difficult to answer, but it is only by hearing from women themselves that we can really understand about women's health and experiences in life.
In case you ever hear of another woman who needs help, here is a list of organizations that provide support, legal advice and counseling services to women in Mwanza. Please do contact them if you or any of your friends or relatives need help. Their services are free, and they will keep anything that anyone says to them private.
