# Supplementary material for: Women’s income and risk of intimate partner violence: secondary findings from the MAISHA cluster randomised trial in North-Western Tanzania
Source: BMC Public Health. 2019 Aug 14;19:1108. doi: 10.1186/s12889-019-7454-1 (PMC6694529; doi:10.1186/s12889-019-7454-1)
Supplement: Supplementary file 3 — Exposure, outcome, contextual and pathway variables. Details of question items used to construct all exposure, outcome, contextual and pathway variables, and how each variable was constructed and coded. (DOCX 17 kb) [file 12889_2019_7454_MOESM3_ESM.docx]

**Additional file 3: Exposure, outcome, contextual and pathway variables**

|  | Indicator | Variable construction |
| --- | --- | --- |
| **Outcome variables** |  |  |
|  | Past year physical IPV | Binary: Yes; No  Coded as ‘Yes’ if she reports that a partner has done at least one of the following things to her in the past year:   - Slapped her or thrown something at her that could hurt her - Pushed her or shoved her or pulled her hair - Hit her with his fist or something else that could hurt her - Kicked her, dragged her or beat her up - Choked or burnt her on purpose - Threatened to use or actually used a gun, knife or other weapon against her |
|  | Past year sexual IPV | Binary: Yes; No  Coded as ‘Yes’ if she reports that any of the following have happened to her in the past year:   - A husband/partner forced her to have sexual intercourse by threatening her, holding her down or hurting her in some way - She had sexual intercourse when she did not want to because she was afraid that her partner would hurt her or someone she cared about if she refused - She had sexual intercourse when she did not want to because she was afraid that her partner would leave her or take another girlfriend if she refused |
|  | Past year economic abuse by a partner | Binary: Yes; No  Coded as ‘Yes’ if she reports that in the past 12 months it is generally true that her partner:   - Refuses to give her money for household expenses, even when he has money for other things - Take money that she has earned away from her - Makes important financial decisions without consulting her |
| **Exposure variables** |  |  |
|  | Monthly income quartile | Categorical: Doesn’t earn; 1^st^ quartile; 2^nd^ quartile; 3^rd^ quartile; 4^th^ quartile; Don’t know  Based on reported daily, weekly or monthly income in Tanzanian Shillings. Reported daily and weekly earnings were converted to monthly earnings on the assumption that each participant worked for 22 days per month. A higher quartile indicates higher income. |
|  | Financial contribution to household relative to husband’s | Binary: Same/less than husband; More than husband  Question: “Would you say that the money that you bring into the household is more than what your husband/partner contributes, less than what he contributes, or about the same as he contributes?” (Response options: Less than partner; Same as partner; More than partner) |
| **Contextual variables** |  |  |
| Respondent demographics | Age | Categorical: <30 years; 30-39 years; 40-49 years; 50+ years |
|  | Highest level of schooling completed | Categorical: None/incomplete primary; Completed primary; Attended secondary or higher |
|  | Believes man should be the primary provider for the family | Binary: Agrees; Disagrees  Respondent asked how much they personally agree or disagree with the following statement: “It must be the man who is the primary provider for the family.” (Response options: I strongly agree; I agree; I disagree; I strongly disagree) |
|  | Witnessed violence against a parent/household member in the home as a child | Binary: Yes (once or more); No (never)  Respondent asked: “Did you see or hear a parent or household member in your home being slapped, kicked, punched or beaten with a fist or object?” (Response options: Never; Once; Few times; Many times) |
| Partner’s demographics | Partner’s age | Categorical: <40 years; 40-49 years; 50+ years |
|  | Highest level of schooling partner completed | Categorical: Completed primary or below/Don’t know; Above primary |
|  | How often respondent has seen partner drunk in the past year | Categorical: Never/Partner doesn’t drink; Once/Few times; Many times  Respondent asked: “In the past 12 months, how often have you seen your partner intoxicated (drunk)?” |
| Relationship | Relationship duration | Categorical: <5 years; 5-9.99 years; 10+ years |
| Household | Household economic hardship in past year | Categorical: Yes; No  Respondent was asked a series of questions:  During the last 12 months…   1. how many times were you very worried/stressed about your general financial situation? 2. have you had trouble buying food or other necessities for your family 3. have you had to borrow money to pay rent or other bills? 4. did any of your family members need to see a doctor but could not because you did not have enough money? 5. did your children miss days of school because you did not have money for school fees, uniform or supplies? 6. Have you or any of your children gone a whole day without eating anything because there was not enough food?   (Response options: Never; Once; Few times; Many times)  Respondents were coded as having experienced household-level hardship in the past year if they answered:   - ‘A few times’ or ‘Many times’ to (a)   *and*   - either:   - ‘A few times’ or ‘Many times’ to any of (b)-(f)   *or*   - - ‘Once’ to at least two of (b)-(f) |
| **Pathway variables** |  |  |
| Arguments with partner | Argue with partner about respondent not fulfilling her responsibilities as wife and mother | Binary: Few/Many times; Never/Once  Respondent asked: “In your relationship with your current/most recent partner, how often have you quarrelled in the past 12 months about accusations that you are not fulfilling your responsibilities as wife and mother?” (Response options: Never; Once; Few times; Many times) |
|  | Argue with partner about his inability or unwillingness to provide for the family | Binary: Few/Many times; Never/Once  Respondent asked: “In your relationship with your current/most recent partner, how often have you quarrelled in the past 12 months about his inability or unwillingness to provide for the family?” (Response options: Never; Once; Few times; Many times) |
|  | Argue with partner about other issues around money and division of resources in the family | Binary: Few/Many times; Never/Once  Respondent asked: “In your relationship with your current/most recent partner, how often have you quarrelled in the past 12 months about other issues around money and division of resources in the family?” (Response options: Never; Once; Few times; Many times) |
| Communication with partner/relationship dynamics | Good communication with partner | Binary: Yes; No  Respondent asked: “During the last 12 months, did you and your partner discuss the following topics together:   - Things that happened to you during the day? - Things that happened to him in the day? - Your worries or feelings? - His worries or feelings?   (Response options: Never; Once; Few times; Many times)  Coded as ‘Yes’ if discussed all of these at least ‘a few times’. Otherwise coded as ‘No’. |
|  | Asked advice by partner in past year | Binary: Few/Many times; Never/Once  Respondent asked: “During the last 12 months, did your partner ever ask you for your advice to resolve a problem he was facing?” (Response options: Never; Once; Few times; Many times) |
|  | Appreciated by partner in past year | Binary: Few/Many times; Never/Once  Respondent asked: “During the last 12 months, did your partner ever make you feel appreciated?” (Response options: Never; Once, Few times; Many times) |
|  | Confidence to asset an opinion if different to partner’s | Binary: Very confident; Not very confident  Respondent asked: How confident do you feel to assert your own opinion if it is different from that of your husband?” (Response options: Very confident; Confident but would need to be encouraged; Not confident at all; Don’t know) |
| Relationship dissolution | Separated during course of study  (among those married or living as married at baseline) | Binary: Yes; No  Respondent reports either:   - not being married/living as married at follow-up   *or*   - is married/living as married at follow-up, but answers ‘no’ to the question “Is this the same man as when we interviewed you two years ago?” |
