# Supplementary material for: Women’s income and risk of intimate partner violence: secondary findings from the MAISHA cluster randomised trial in North-Western Tanzania
Source: BMC Public Health. 2019 Aug 14;19:1108. doi: 10.1186/s12889-019-7454-1 (PMC6694529; doi:10.1186/s12889-019-7454-1)
Supplement: Supplementary file 4 — a Percentage of women in each income category who experienced ‘pathway’ factors in past year (baseline). b Percentage of women in each relative financial contribution category who experienced ‘pathway’ factors in past year (baseline). Tables of baseline associations between income indicators and potential ‘pathway’ factors (DOCX 15 kb) [file 12889_2019_7454_MOESM4_ESM.docx]

Additional file 4a: Percentage of women in each income category who experienced ‘pathway’ factors in past year (baseline), presented with chi-squared p-value for association between income and pathway variable

| Woman’s income quartile | N | Household experienced economic hardship | Partner accuses her of not fulfilling her role as wife/mother | Argues with partner over his unwillingness/inability to provide for family | Argues with partner over other money issues/division of resources in family | Good communication with partner | Very confident to assert opinion if different from husband's | Husband asks her advice to resolve problems he is facing | Husband makes her feel appreciated | Separated from partner between baseline and follow-up |
| --- | --- | --- | --- | --- | --- | --- | --- | --- | --- | --- |
| Doesn't earn | 31 | 39% | 35% | 19% | 23% | 45% | 48% | 65% | 87% | 6% |
| 1st quartile | 206 | 48% | 35% | 44% | 34% | 41% | 43% | 67% | 77% | 9% |
| 2nd quartile | 199 | 46% | 32% | 36% | 34% | 46% | 53% | 66% | 78% | 11% |
| 3rd quartile | 145 | 34% | 30% | 34% | 29% | 57% | 58% | 81% | 89% | 12% |
| 4th quartile | 132 | 33% | 26% | 33% | 34% | 58% | 60% | 81% | 86% | 14% |
| Don't know | 27 | 44% | 22% | 44% | 44% | 22% | 44% | 56% | 70% | 33% |
| chi-squared p-value |  | 0.025 | 0.469 | 0.054 | 0.494 | 0.000 | 0.027 | 0.001 | 0.013 | 0.009 |
| chi-squared p-value, excluding don't know and don’t' earn |  | 0.005 | 0.349 | 0.110 | 0.738 | 0.002 | 0.008 | 0.001 | 0.009 | 0.513 |

Additional file 4b: Percentage of women in each relative financial contribution category who experienced ‘pathway’ factors in past year (baseline), presented with chi-squared p-value for association between relative financial contribution and pathway variable

| Woman’s financial contribution to household | N | Partner accuses her of not fulfilling her role as wife/mother | Argues with partner over his unwillingness/inability to provide for family | Argues with partner over other money issues/division of resources in family | Good communication with partner | Very confident to assert opinion if different from husband's | Husband asks her advice to resolve problems he is facing | Husband makes her feel appreciated | Separated from partner between baseline and follow-up |
| --- | --- | --- | --- | --- | --- | --- | --- | --- | --- |
| Same/less than husband | 533 | 29% | 31% | 29% | 52% | 51% | 78% | 87% | 9% |
| More than husband | 207 | 36% | 53% | 43% | 37% | 54% | 55% | 67% | 19% |
| chi-squared p-value |  | 0.097 | <0.001 | <0.001 | <0.001 | 0.48 | <0.001 | <0.001 | <0.001 |
