# Supplementary material for: Women’s income and risk of intimate partner violence: secondary findings from the MAISHA cluster randomised trial in North-Western Tanzania
Source: BMC Public Health. 2019 Aug 14;19:1108. doi: 10.1186/s12889-019-7454-1 (PMC6694529; doi:10.1186/s12889-019-7454-1)
Supplement: Supplementary file 5 — Forest plots of odds ratios of association between pathway variables and past year physical IPV, sexual IPV and economic abuse (DOCX 30 kb) [file 12889_2019_7454_MOESM5_ESM.docx]

Additional file 5: Forest plots of odds ratios of association between pathway variables and past year physical IPV, sexual IPV and economic abuse
